# Enhancing intraneural revascularization following peripheral nerve injury through hypoxic Schwann-cell-derived exosomes: an insight into endothelial glycolysis

**DOI:** 10.1186/s12951-024-02536-y

**Published:** 2024-05-24

**Authors:** Jun Sun, Qiuhua Zeng, Zhimin Wu, Zhangyu Li, Qun Gao, Zhi Liao, Hao Li, Cong Ling, Chuan Chen, Hui Wang, Baoyu Zhang

**Affiliations:** 1 Department of Neurosurgery, the Third Affiliated Hospital of Sun Yat-sen University, No. 600 Tianhe Road, Guangzhou, Guangdong 510630 PR China; 2 Department of Radiology, Guangdong Provincial Hospital of Chinese Medicine, Guangzhou, 510000 China; 3 Department of Neurosurgery, School of Medicine, the First Affiliated Hospital of Xiamen University, Xiamen University, Xiamen, 361102 China; 4 Department of Neurosurgery, Peking University People’s Hospital, 11th Xizhi Men South St, Beijing, 100044 China; 5 Department of Neurosurgery, Guangzhou Panyu Central Hospital, No.8, Fuyu East Road, Qiaonan Street, Panyu District, Guangzhou, 511400 Guangdong PR China

**Keywords:** Exosome, Glycolysis, Intraneural revascularization, miR-21-5p, Peripheral nerve injury, Sciatic nerve, Schwann cell

## Abstract

**Background:**

Endothelial cell (EC)-driven intraneural revascularization (INRV) and Schwann cells-derived exosomes (SCs-Exos) both play crucial roles in peripheral nerve injury (PNI). However, the interplay between them remains unclear. We aimed to elucidate the effects and underlying mechanisms of SCs-Exos on INRV following PNI.

**Results:**

We found that GW4869 inhibited INRV, as well as that normoxic SCs-Exos (N-SCs-Exos) exhibited significant pro-INRV effects in vivo and in vitro that were potentiated by hypoxic SCs-Exos (H-SCs-Exos). Upregulation of glycolysis emerged as a pivotal factor for INRV after PNI, as evidenced by the observation that 3PO administration, a glycolytic inhibitor, inhibited the INRV process in vivo and in vitro. H-SCs-Exos more significantly enhanced extracellular acidification rate/oxygen consumption rate ratio, lactate production, and glycolytic gene expression while simultaneously suppressing acetyl-CoA production and pyruvate dehydrogenase E1 subunit alpha (PDH-E1α) expression than N-SCs-Exos both in vivo and in vitro. Furthermore, we determined that H-SCs-Exos were more enriched with miR-21-5p than N-SCs-Exos. Knockdown of miR-21-5p significantly attenuated the pro-glycolysis and pro-INRV effects of H-SCs-Exos. Mechanistically, miR-21-5p orchestrated EC metabolism in favor of glycolysis by targeting von Hippel-Lindau/hypoxia-inducible factor-1α and PDH-E1α, thereby enhancing hypoxia-inducible factor-1α-mediated glycolysis and inhibiting PDH-E1α-mediated oxidative phosphorylation.

**Conclusion:**

This study unveiled a novel intrinsic mechanism of pro-INRV after PNI, providing a promising therapeutic target for post-injury peripheral nerve regeneration and repair.

**Graphical Abstract:**

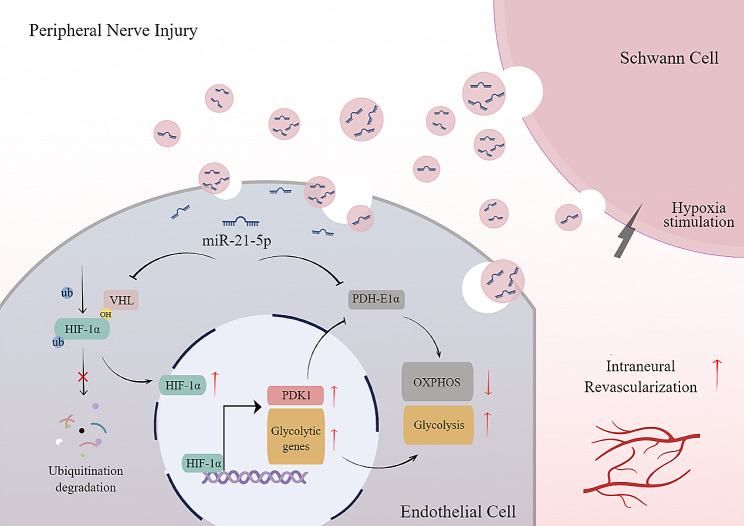

**Supplementary Information:**

The online version contains supplementary material available at 10.1186/s12951-024-02536-y

## Background

The peripheral nervous system (PNS), a multifaceted entity encompassing axons, myelin sheaths, and intraneural vessels coursing within nerves, is a renewable tissue in adult mammals following injury [1]. Despite the superior regenerative potential of the PNS when juxtaposed with the non-regenerative central nervous system (CNS), peripheral nerve injury (PNI) often leads to unsatisfactory structural and functional recovery owing to the sluggish pace and efficiency of regeneration, frequently resulting in partial or complete loss of sensory, motor, and autonomic functions [2, 3].

The vascular network constitutes an indispensable component of the PNS, playing a pivotal role in maintaining the dynamic equilibrium of the nervous system microenvironment and facilitating neural structural repair, development, and functional restoration [4–10].

Following PNI, the regeneration of intraneural vessels often precedes the regeneration of axons and myelin sheaths. The initial formation of polarized vessels not only serves as a navigational scaffold for Schwann cell (SC) bundles and axons but also as a source of energy and nutrition. This scaffold aids in directing SC bundle and axon migration and regeneration from the proximal to the distal end of the severed sciatic nerve [10–12]. Notably, the accurate emulation of the natural vascular system remains a formidable challenge, and the underlying intrinsic mechanisms that trigger native intraneural vessel reconstruction after PNI remain enigmatic.

Endothelial cells (ECs) lining the luminal surface of blood vessels typically maintain a quiescent state in mature blood vessels to preserve vascular homeostasis. However, they transform into an active pro-angiogenic state in response to injury. The physiological function of ECs is intimately tied to their specific metabolic pattern, with 80% of their energy supplied via glycolytic metabolism [13–15]. Alterations in the expression of key glycolytic enzymes, such as PFKFB3 or GLUT1, significantly impact the angiogenic capacity of ECs [16–19]. Despite this knowledge, our understanding of whether and how changes in the glycolytic metabolism of vascular ECs affect polarized vessel formation in the PNS remains limited.

As the glial cells of the PNS, SCs wield substantial influence over post-injury regenerative processes by facilitating axonal regrowth, elongation, and remyelination through diverse pathways [20]. A recent single-cell sequencing study hinted at the existence of a close interaction between SCs and ECs in the PNS. However, the precise roles and underlying mechanisms of this interaction remain obscure [21]. Exosomes, small bi-lipid membrane vesicles measuring 50 to 200 nm in diameter and containing DNA, mRNA, miRNA, proteins, and other cellular components, play a pivotal role in mediating intercellular communication, including tissue angiogenesis [22, 23]. Intriguingly, exosomes are also implicated in the regulation of intercellular energy metabolism [24, 25]. Thus, we postulated that exosomes derived from SCs (SCs-Exos) may be associated with EC-mediated post-injury intraneural revascularization by modulating energy metabolism.

In this study, we present evidence that SCs-Exos enhances the glycolytic metabolism of ECs, thereby facilitating intraneural revascularization, axon regeneration, and functional recovery following PNI. Furthermore, we elucidate how the upregulation of miR-21-5p in SCs-Exos following hypoxic preconditioning strengthens this metabolic reprogramming by targeting the von Hippel-Lindau/hypoxia-inducible factor-1α (VHL/HIF-1α) and the pyruvate dehydrogenase-E1α subunit (PDH-E1α) pathways simultaneously.

## Results

### Exosomal shuttle-mediated intraneural revascularization after PNI

**Fig. 1:**
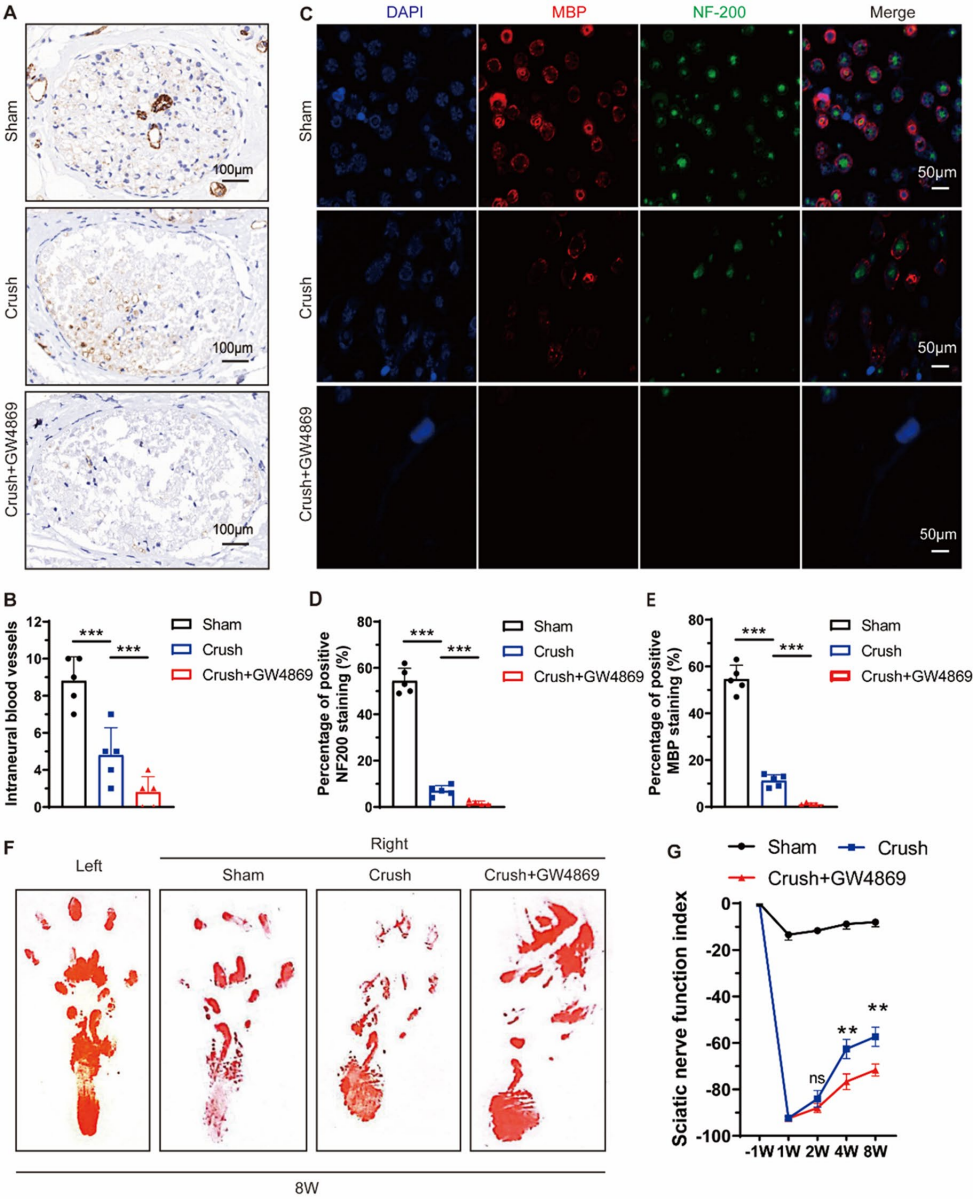
Exosomal shuttle is involved in post-injury intra-neuro-revascularization and nerve repair after sciatic nerve injury. (**A**). Representative immunohistochemical staining of CD31 (brown) and nuclei (blue) was conducted to visualize intraneural blood vessels in the sciatic nerve segments from both the sham group (exposed sciatic nerve without injury) and the crush group (with crush injury), with or without administration of GW4869 (scale bar = 100 μm, *n* = 5 rats/group). (**B**). The quantifications of the numbers of intraneural blood vessels shown in (**A**). (**C**). Representative immunofluorescent staining of neurofilaments (NF-200, green), myeline (MBP, red), and nuclei (DAPI, blue) to evaluate post-injury nerve regeneration (scale bar = 50 μm, *n* = 5 rats/group). (**D-E**). The quantifications of the NF-200 and MBP positive area shown in (C). (**F**). Representative footprints of rats with or without GW4869 administration after 8 weeks of sciatic nerve crush injury. The left image shows the control group and the right image the sham or operative group. (**G**). Sciatic functional index (SFI) analysis to evaluate functional recovery from weeks 1 to 8 after sciatic nerve injury (*n* = 5 rats/group). ^*^P < 0.05, ^**^P < 0.01, ^***^P < 0.001. Bars represent group means ± SD. A Student’s *t* test was used for comparisons. NF-200: Neurofilament-200; MBP: Myelin basic protein

According to previous studies, the angiogenic process typically occurs between the first and fifth day following PNI [12], In our investigation, we collected nerve segments on the fifth day post-injury for further analysis. The immunohistochemistry (IHC) results revealed that the administration of GW4869, an inhibitor of exosome release, led to a significant reduction in CD31 + blood vessels within the sciatic nerve, indicating a substantial inhibition of intraneural revascularization (Fig. 1A and B). This observation aligns with previous research emphasizing the role of exosomes in regulating nerve regeneration and repair [26, 27]. Notably, GW4869 administration also hindered axonal (anti-NF-200) and myelin (anti-MBP) regeneration (Fig. 1C and E). Furthermore, our assessment of paw print patterns and the sciatic nerve function index (SFI) demonstrated delayed recovery in both sensory and motor functions (Fig. 1F and G). These findings collectively suggest a close relationship between exosomal transport within the injured sciatic nerve and intraneural revascularization, post-injury axon regeneration, and functional recovery following PNI.

### SCs-Exos facilitated intraneural revascularization and hypoxic precondition enhanced this process

To explore whether SCs-Exos affected angiogenesis in vitro, we collected the culture supernatants from SCs subjected to normoxic or hypoxic conditions (N-SCs supernatants or H-SCs supernatants) for co-culturing with human umbilical vein endothelial cells (HUVECs). Our results showed that N-SCs-supernatants facilitated HUVEC tube formation (Fig. S1A and S1D), invasion (Fig. S1B and S1E), and proliferation (Fig. S1C and S1F). Interestingly, H-SCs supernatants appeared to have a stronger pro-angiogenic role than N-SCs supernatants (Fig. S1A–S1F). To confirm the involvement of exosomes in pro-revascularization in vivo, we employed GW4869 to inhibit exosome secretion. Our results revealed that the pro-vascular effects of both N-SCs supernatants and H-SCs supernatants were effectively counteracted by GW4869 treatment (Fig. S1A–S1F). Subsequently, we established a co-culture system to further investigate the impact on dorsal root ganglion neurons (DRGns; Fig. S2A). Furthermore, our findings demonstrated that ECs treated with H-SCs supernatants exhibited greater promotion of axonal growth compared to those treated with N-SCs supernatants. However, GW4869 administration mitigated the effects of both types of supernatants (Fig. S2B and S2C). These results suggest that SCs-Exos mediate the pro-angiogenic role of SCs in ECs, further enhancing neural axonal growth in vitro. Notably, hypoxic preconditioning amplifies this positive effect.

**Fig. 2:**
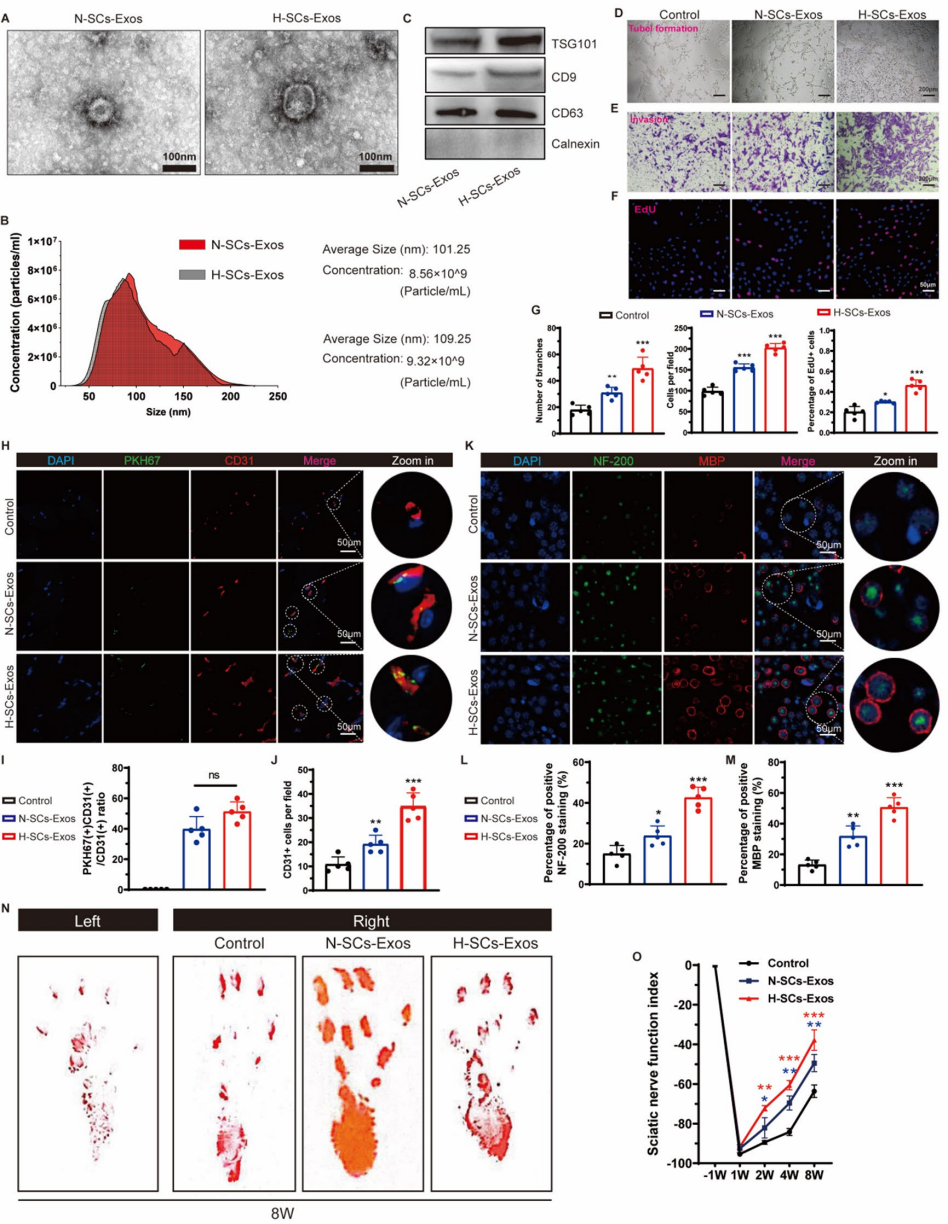
H-SCs-Exos promotes intraneural angiogenesis and nerve repair after sciatic nerve injury. (**A**). Representative TEM for N-/H-SCs-Exos (scale bar = 100 μm). (**B**). NTA for concentration and size of N-/H-SCs-Exos. (**C**). Western blot assay for detecting exosomal-specific markers TSG101, CD9, and CD63 and cytoplasm marker calnexin. (**D–F**). Representative tube formation, transwell invasion, and EdU assay for evaluating endothelial angiogenic capacity after N-/H-SCs-Exos treatment (scale bar = 200–50 μm). (**G**). Data analysis for D to F (*n* = 5 rats/group). (**H**). PKH-67-labeled N-H-SCs-Exos (green) track in intraneural endothelial cells (ECs; CD31+, red) of injured sciatic nerve sites (scale bar = 50 μm, *n* = 5 rats/group). (**I**). CD31 + PKH-67+/CD31 + ratio. (**J**). CD31 + ECs per field analysis. (**K**). Representative immunofluorescent staining of neurofilaments (NF-200, green), myeline (MBP, red), and nuclei (DAPI, blue) to evaluate post-injury nerve regeneration (scale bar = 50 μm, *n* = 5 rats/group) and data analysis shown in **L** to **N.** Representative footprints of rats with saline or N-/H-SCs-Exos administration at 8 weeks after sciatic nerve crush injury. The left image shows the control group and the right image the sham or operative group. (**O**). Sciatic functional index calculation to evaluate functional recovery from week 1 to 8 after sciatic nerve injury (*n* = 5 rats/group). ^*^P < 0.05, ^**^P < 0.01, ^***^P < 0.001. Bars represent group means ± SD. A Student’s *t* test was used for comparisons. NF-200: Neurofilament-200; MBP: Myelin basic protein; TEM: Transmission electron microscope; NTA: Nanosight tracking analysis; SFI: Sciatic functional index

We next employed ultracentrifugation to isolate exosomes from the supernatants of SC cultures, adhering to established protocols detailed in prior publications [28, 29]. Transmission electron microscopy (TEM) analysis revealed that both N-SCs-Exos and H-SC-Exos exhibited characteristic bilayered, spherical membrane vesicles (Fig. 2A). The size of N-SCs-Exos and H-SCs-Exos spanned from 30 to 212 nm (average 101.25 nm) and 40 to 225 nm (average 109.25 nm), respectively, and had a concentration of 8.56 × 10^9^ and 9.32 × 10^9^ particles/ml, respectively (Fig. 2B). Western blot analysis confirmed the positive expression of exosomal-specific markers, including CD9, CD63, and TSG101. Notably, the cytoplasmic protein calnexin was not detected in exosomes (Fig. 2C). These data suggest that the exosomes from SCs were successfully purified.

For the in vitro investigation, we co-cultured the two purified exosomes with HUVECs. The results showed that the H-SCs-Exos significantly promoted HUVEC tube formation (Fig. 2D and G), invasion (Fig. 2E and G), and proliferation (Fig. 2F and G). Further, a co-culture system of ECs and DRGns was established (Fig. S2D), and H-SCs-Exos-treated ECs more strongly promoted DRGns axonal growth (Fig. S2E). To determine the precise effects of N-SCs-Exos and H-SCs-Exos on intraneural endothelial cells in vivo, PKH-67-labeled N-SCs-Exos, and H-SCs-Exos were locally injected into the sciatic nerve injury site. The co-localized expression of PKH-67 + CD31 + was detected to evaluate the internalized capacity of vascular endothelial cells in the sciatic nerve injury site to two types of exosomes. The observed internalization of exosomes revealed that the intraneural ECs had great internal ability to both N-SCs-Exos and H-SCs-Exos and that the intensity of H-SCs-Exos is slightly higher than that of N-SCs-Exos (Fig. 2H and I). Consistent with the in vitro results, H-SCs-Exos facilitated intraneural revascularization (CD31 + blood vessels) more strongly than N-SCs-Exos (Fig. 2H and J).

We then aimed to further ascertain the post-injury structural construction of sciatic nerves. IF for detection of NF-200 + neurofilament and MBP + myelin showed that both N-SCs-Exos and H-SCs-Exos strongly enhanced the fluorescent intensity and that H-SCs-Exos did so more strongly (Fig. 2K and M). In the detection of SFI, we found that both N-SCs-Exos and N-SCs-Exos significantly improved sciatic nerve function and that H-SCs-Exos did so more strongly (Fig. 2N and O). These data suggest that the localized hypoxic precondition enhances the promotion of SCs-Exos in intraneural revascularization, axon regeneration, and functional recovery.

### SCs-Exos reprogram endothelial energy metabolism to facilitate intraneural revascularization

The endothelial glycolysis level is intimately associated with post-injury revascularization. To determine the role of endothelial metabolic changes in post-injury intraneural revascularization, we detected the metabolites at the injury site of the sciatic nerve. The results showed noticeably increased lactate (Fig. S2A) and pyruvate (Fig. S2B) production and decreased acetyl-coenzyme A (acetyl-CoA) production (Fig. S2C) after spinal cord injury (SCI), whereas GW4869 administration counteracted this metabolic reprogramming (Fig. S2A–3 C). The IHC results showed lactate dehydrogenase A (LDHA) conversion of pyruvate to lactate in both the entire nerve section and intraneural blood vessels had increased (Fig. S3D–3 F), whereas pyruvate dehydrogenase-E1-alpha subunit (PDH-E1α) conversion of pyruvate to acetyl-CoA had decreased (Fig. S3G–I).

**Fig. 3:**
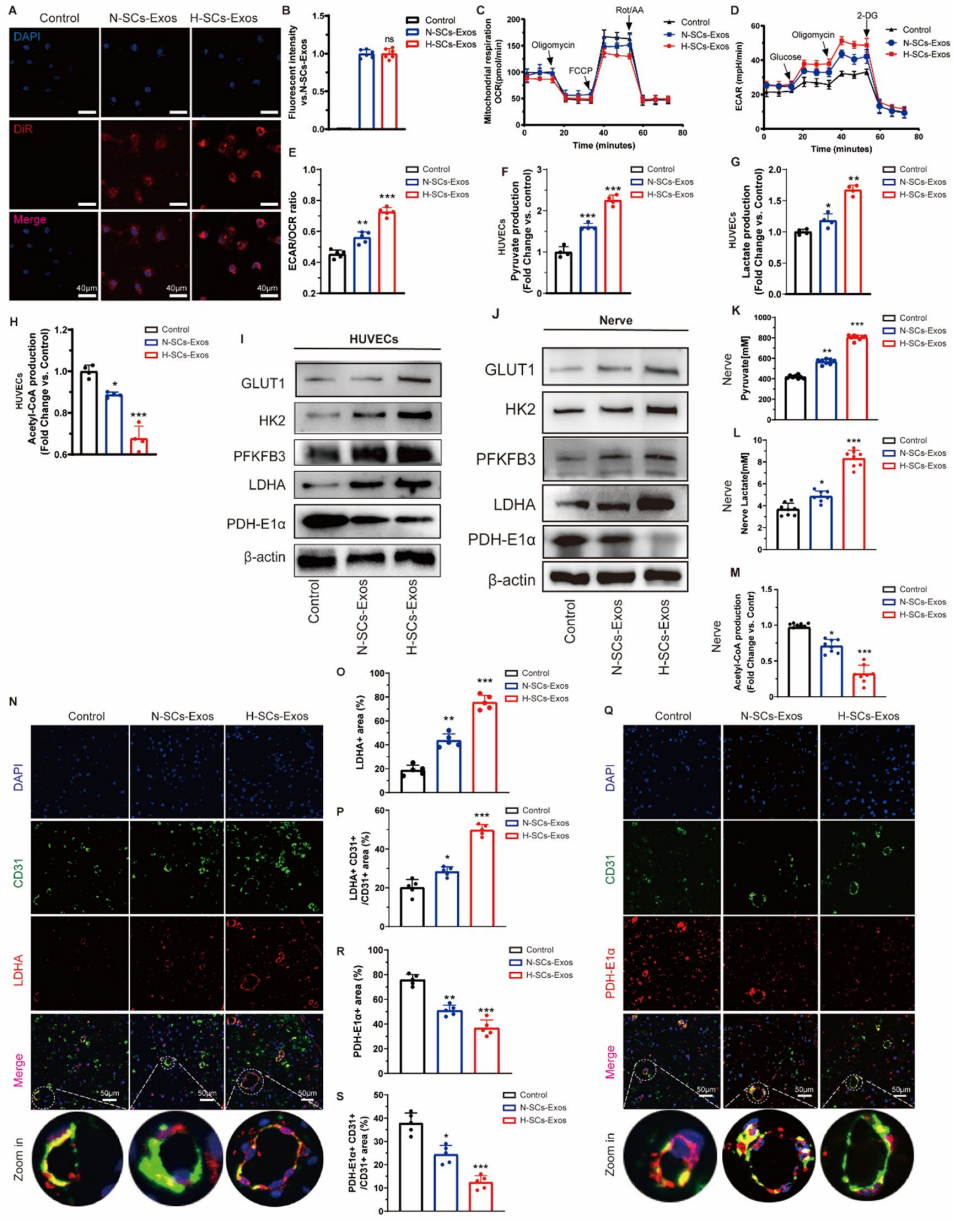
H-SCs-Exos boosts glycolysis but inhibits oxidative phosphorylation in vitro and in vivo. (**A**). Capability of HUVECs to endocytose DiR-labeled N-/H-SCs-Exos (red, DiR; blue, DAPI; scale bar = 40 μm, *n* = 6 rats/group. (**B**). Fluorescent intensity analysis of A. **C.** HUVEC OCR assay for evaluating cellular mitochondrial respiration. (**D**). HUVECs’ ECAR assay for evaluating cellular glycolytic level. (**E**). ECAR/OCR ratio calculation for further analysis. (**F**). Pyruvate production, (**G**). lactate production, and (**H**). acetyl-coA production of HUVECs after N-/H-SCs-Exos treatment (*n* = 4 rats/group). (**I**). GLUT1, HK2, PFKFB3, LDHA, and PDH-E1α protein expression in HUVECs after N-/H-SCs-Exos treatment. β-actin was used as the reference protein (*n* = 5 rats/group). (**J**). GLUT1, HK2, PFKFB3, LDHA, and PDH-E1α protein expression in injured sciatic nerve segments after N-/H-SCs-Exos treatment. β-actin was used as the reference protein (*n* = 5 rats/group). (**K**). Pyruvate production, (**L**). lactate production, and (**M**). acetyl-coA production of HUVECs after N-/H-SCs-Exos treatment (*n* = 8 rats/group). (**N**–**P**). Representative immunofluorescent staining of intraneural ECs (CD31+, green), LDHA (red), and nuclei (DAPI, blue) to evaluate glycolysis changes of entire injured sciatic nerve site and intraneural ECs after N-/H-SCs-Exos treatment (scale bar = 50 μm, *n* = 5 rats/group). (**Q**–**S**). Representative immunofluorescent staining of intraneural ECs (CD31+, green), PDH-E1α (red), and nuclei (DAPI, blue) to evaluate changes in pyruvate production and mitochondrial tricarboxylic acid cycle (TCA) metabolism at entire injured sciatic nerve site and intraneural ECs after N-/H-SCs-Exos treatment (scale bar = 50 μm, *n* = 5 rats/group). ^*^P < 0.05, ^**^P < 0.01, ^***^P < 0.001. Bars represent group means ± SD. A Student’s *t* test was used for comparisons. HUVECs: Human umbilical vein endothelial cells; GLUT1: Glucose transport 1; PFKFB3: 6-phosphofructo-2-kinase/fructose-2,6-bisphosphatase 3; HK2: Hexokinase 2; LDHA: Lactate dehydrogenase A; PDH-E1α: Pyruvate dehydrogenase E1 alpha 1; ECAR: Extracellular acidification rate; OCR: Oxygen consumption rate

Consistently, the GW4869 administration reversed the aforementioned processes (Fig. S3G–I). The results suggest that crush injury shifts the metabolic phenotype of local sciatic nerves in favor of glycolysis. To further determine the effects of this metabolic shift on post-injured nerve repair, 3PO, a specific inhibitor of the glycolytic pathway, was administered. The results revealed that glycolysis inhibition by 3PO significantly hampered the nerve fiber (anti-NF200) and myelin reconstruction (anti-MBP; Fig. S4A–C). The SFI results showed that 3PO delayed the automatic repair process of sciatic nerve function (Fig. S3D and S3E). Collectively, our data indicate that exosomal shuttling is involved in injury-triggered metabolic reprogramming of endothelial cells in favor of glycolysis to facilitate intraneural revascularization and further functional recovery.

SCs-Exos promotion of endothelial angiogenesis via regulation of metabolic reprogramming was next explored. First, both DiR-labeled N- and H-SCs-Exos were well internalized by HUVECs without significant differences (Fig. 3A and B). Observation of the slight predominance of internalization of H-SCs-Exos in vivo indicates that the main reason for the lack of differences was the sufficient concentration of exosomes, which masked the differences in vitro. The extracellular acid rate (ECAR) and oxygen consumption rate (OCR) results showed that H-SCs-Exos more significantly enhanced the ECAR (Fig. 3C) and inhibited the OCR of HUVECs (Fig. 3D), increasing the ECAR/OCR ratio (Fig. 3E), which indicated upregulated glycolytic metabolism and downregulated mitochondrial respiration.

The metabolites pyruvate, lactate, and acetyl-CoA were detected in HUVECs, of which H-SCs-Exos more strongly increased the production of pyruvate (Fig. 3F) and lactate (Fig. 3G) but more strongly decreased the production of acetyl-CoA (Fig. 3H) than N-SCs-Exos. Finally, the western blotting results showed that H-SCs-Exos more strongly upregulated the expression of the glycolytic enzyme protein than N-SCs-Exos, including glucose transporter (GLUT1), hexokinase2 (HK2), 6-phosphofructo-2-kinase/fructose-2,6-bisphosphatase 3 (PFKFB3) and LDHA (Fig. 3I), while downregulated PDH-E1α, which mediates acetyl-coA production (Fig. 3I).

Western blot analysis after N- or H-SCs-Exos were locally administered in vivo showed that H-SCs-Exos administration more significantly increased the overall level of the lactate-produced protein enzymes CLUT1, HK2, PFKFB3, and LDHA at the injury site after SCI than N-SCs-Exos administration (Fig. 3J). In contrast, the PDH-E1α level was more strongly decreased by H-SCs-Exos administration (Fig. 3J). The metabolite assays showed that H-SCs-Exos administration more strongly increased pyruvate (Fig. 3K) and lactate (Fig. 3L) production than N-SCs-Exos administration. In contrast, H-SCs-Exos administration more significantly decreased acetyl-CoA production (Fig. 3M).

The immunofluorescence (IF) results of LDHA + CD31+ /CD31 + ratio and PDH-E1α + CD31 + cells/CD31 + ratio analysis showed that H-SCs-Exos administration more significantly increased the expression of LDHA in both the entire injured nerve (Fig. 3N and O) and intraneural ECs (Fig. 3N and P) than N-SCs-Exos administration but decreased PDH-E1α production (Fig. 3Q and S). Considered together, these results suggest that hypoxic preconditioning enhances the function of SCs-Exos in upregulating glycolysis and downregulating oxidative phosphorylation (OXPHOS) of intraneural vascular endothelial cells to facilitate intraneural revascularization and nerve repair.

### Mir-21-5p transfer is responsible for the metabolic regulation of SCs-Exos on endothelial cells

MicroRNAs are enriched in exosomes and perform various biological functions [30]. According to our studies and those of other authors, SCs and their released exosomes are enriched with miR-21-5p [26, 31, 32]. Interestingly, in the present study, H-SCs-Exos were more enriched with miR-21-5p than N-SCs-Exos (Fig. S5A), in agreement with the characteristics of miR-21-5p as a hypoxia-responsive miRNA, as previously reported [33]. Both pri-miR-21-5p and pre-miR-21-5p quantities in the nerve after SCI strongly increased (Fig. S5B and S5C). Further, after local administration with N-SCs-Exos or H-SCs-Exos, the pre-miR-21-5p quantity at the injury site of the sciatic nerve was more strongly increased by H-SCs-Exos than by N-SCs-Exos administration, while neither affected pri-miR-21-5p quantity (Fig. S5D and S5E). Similarly, the pre-miR-21-5p quantity in HUVECs treated with H-SCs-Exos was higher than that of those treated with N-SCs-Exos or phosphate-buffered saline (PBS, ) while the pri-miR-21-5p quantity did not change (Fig. S5F and S5G). Our data suggest that miR-21-5p could be transferred rather than stimulated by both N-SCs-Exos and H-SCs-Exos to ECs and that H-SCs-Exos are more strongly enriched with miR-21-5p.

**Fig. 4:**
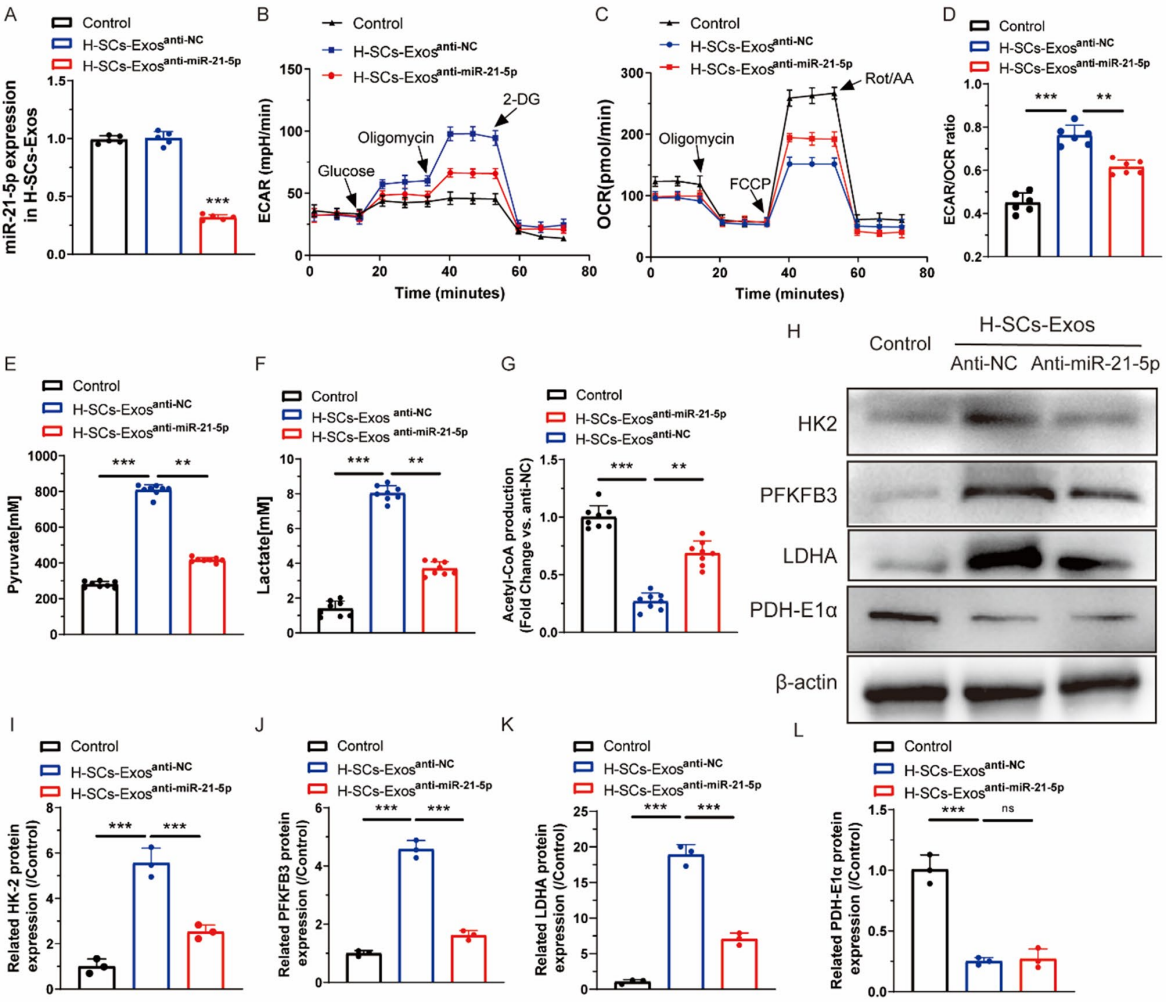
H-SCs-Exos increase glycolysis of ECs through transferring miR-21-5p in vitro. (**A**). qRT-PCR assay for expression of miR-21-5p in H-SCs-Exos after treatment with PBS (control), anti-NC, or anti-miR-21-5p (*n* = 5 rats/group). (**B**). HUVEC ECAR assay for evaluating cellular glycolytic level (*n* = 6 rats/group). (**C**). HUVEC OCR assay for evaluating cellular mitochondrial respiration (*n* = 6 rats/group). (**D**). ECAR/OCR ratio calculation for further analysis (*n* = 6 rats/group). (**E**). Pyruvate production, (**F**). lactate production, and (**G**). acetyl-coA production of HUVECs after N-/H-SCs-Exos treatment (*n* = 8/group). (**H–L**). Western blot showing protein expression of HK2, PFKFB3, LDHA, and PDH-E1α in HUVECs after PBS, H-SCs-Exos^anti−NC^, or H-SCs-Exos^anti−miR−21−5p^ treatment. β-actin was used as the reference protein (*n* = 3 rats/group). ^*^P < 0.05, ^**^P < 0.01, ^***^P < 0.001. Bars represent group means ± SD. A Student’s *t* test was used for comparisons. HUVECs: Human umbilical vein endothelial cells; PFKFB3: 6-phosphofructo-2-kinase/fructose-2,6-bisphosphatase 3; HK2: Hexokinase 2; LDHA: Lactate dehydrogenase A; PDH-E1α: Pyruvate dehydrogenase E1 alpha 1; ECAR: Extracellular acidification rate; OCR: Oxygen consumption rate

To verify whether metabolic regulation of SCs on endothelial cells was through transferring miR-21-5p, H-SCs-Exos were extracted from hypoxia-cultured SCs that had been pretreated with an anti-miR-21-5p oligonucleotide (H-SCs-Exos^anti-miR-21−5p^) or with scrambled control (H-SCs-Exos^anti-NC^; Fig. 4A). Next, the mitochondrial metabolic program was detected by measuring ECAR and OCR, which showed that H-SCs-Exos^anti-NC^-upregulated ECAR of HUVECs was counteracted by H-SCs-Exos^anti-miR-21−5p^ (Fig. 4B). In contrast, the decreased OCR of HUVECs by H-SCs-Exos^anti-NC^ was partly rescued by H-SCs-Exos^anti-miR-21−5p^ (Fig. 4C). The ECAR/OCR ratio of HUVECs notably increased after H-SCs-Exos^anti-NC^ administration, which was counteracted by H-SCs-Exos^anti-miR-21−5p^ administration (Fig. 4D).

Metabolite assay showed that H-SCs-Exos^anti-miR-21−5p^ administration significantly reversed H-SCs-Exos^anti-NC^-increased pyruvate (Fig. 4E) and lactate (Fig. 4F) production and decreased acetyl-CoA (Fig. 4G) production. The western blot results showed that H-SCs-Exos^anti-miR-21−5p^ administration strongly reversed H-SCs-Exos^anti-NC^-upregulated HK2, PFKFB3, and LDHA protein expression, but didn’t recover H-SCs-Exos^anti-NC^-downregulated PDH-E1α protein expression (Fig. 4H and L).

**Fig. 5:**
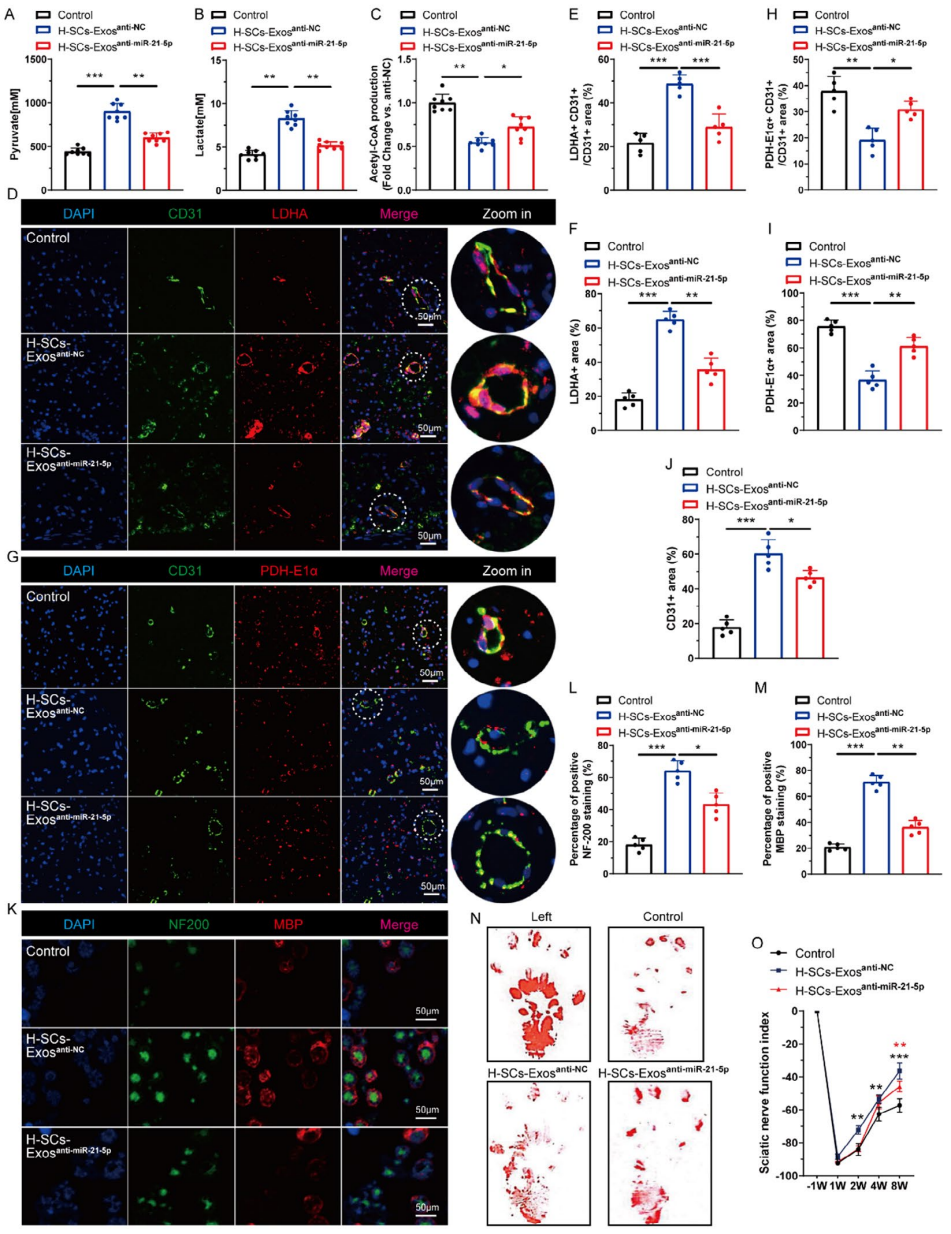
H-SCs-Exos regulate EC energy metabolism programming through transferring miR-21-5p in vivo. (**A**). Pyruvate production, (**B**). lactate production, and (**C**). acetyl-coA production at injured sciatic nerve sites after saline (control), H-SCs-Exos^anti−NC^, or H-SCs-Exos^anti−miR−21−5p^ treatment (*n* = 8 rats/group). (**D–F**). Representative immunofluorescent staining of intraneural ECs (CD31+, green), LDHA (red), and nuclei (DAPI, blue) to evaluate glycolytic metabolism of entire injured sciatic nerve and intraneural ECs (scale bar = 50 μm, *n* = 5 rats/group). (**G-I**). Representative immunofluorescent staining of intraneural ECs (CD31+, green), PDH-E1α (red), and nuclei (DAPI, blue) to evaluate pyruvate production and mitochondrial tricarboxylic acid cycle metabolism of entire injured sciatic nerve and intraneural ECs (scale bar = 50 μm, *n* = 5 rats/group). (**J**). CD31 + vascular endothelial cell (EC) counts in D and G. (**K–M**). Representative immunofluorescent staining of neurofilaments (NF-200, green), myeline (MBP, red), and nuclei (DAPI, blue) to evaluate post-injury nerve regeneration (scale bar = 50 μm, *n* = 5 rats/group. (**N**). Representative footprints of rats with saline, H-SCs-Exos^anti−NC^, or H-SCs-Exos^anti−miR−21−5p^ administration after 8 weeks of sciatic nerve crush injury. The left image shows the control group and the right image sham or operative group. (**O**). Sciatic functional index calculation to evaluate functional recovery from week 1 to 8 after sciatic nerve injury (*n* = 5 rats/group). ^*^P < 0.05, ^**^P < 0.01, ^***^P < 0.001. Bars represent group means ± SD. A Student’s *t* test was used for comparisons. NF-200: Neurofilament-200; MBP: Myelin basic protein; TEM: Transmission electron microscope; NTA: Nanosight tracking analysis; ECAR: Extracellular acidification rate; OCR: Oxygen consumption rate; LDHA: Lactate dehydrogenase A; PDH-E1α: Pyruvate dehydrogenase E1 alpha

In vivo, H-SCs-Exos^anti-miR-21−5p^ administration reversed the H-SCs-Exos^anti-NC^-augmented pyruvate (Fig. 5A) and lactate (Fig. 5B) production and reduced acetyl-CoA (Fig. 5C) production at injury site nerve segments. The IF results for LDHA + CD31 + cells and PDH-E1α + CD31 + cells at the injury site showed that H-SCs-Exos^anti-miR-21−5p^ administration reversed the H-SCs-Exos^anti-NC^-increased percentage of the LDHA + CD31 + area (Fig. 5D and E) and the overall lactate dehydrogenase A (LDHA) expression in injured sciatic nerve segments (Fig. 5D and F). H-SCs-Exos^anti-miR-21−5p^ administration also counteracted the H-SCs-Exos^anti-NC^-decreased percentage of PDH-E1α + CD31 + area (Fig. 5G and H) and the overall PDH-E1α level (Fig. 5G and I). These data suggest that H-SCs-Exos skew endothelial metabolism in favor of glycolysis.

Knockdown of miR-21-5p impeded H-SCs-Exos-initiated intraneural revascularization (Fig. 5G and J) and H-SCs-Exos^anti-miR-21−5p^ administration significantly decreased the promotional effects of H-SCs-Exos^anti-NC^ on axonal regeneration (Fig. 5K and L) and remyelination (Fig. 5K and M). Unsurprisingly, the paw print and SFI results showed that H-SCs-Exos^anti-miR-21−5p^ notably impeded H-SCs-Exos^anti-NC^-enhanced sciatic nerve function (Fig. 5N and O). These data suggest that miR-21-5p transfer is mainly responsible for the significant pro-revascularization and pro-regeneration effects of H-SCs-Exos after PNI.

### Mir-21-5p regulates endothelial metabolic programming via targeting VHL/HIF-1α and PDH-E1α

**Fig. 6:**
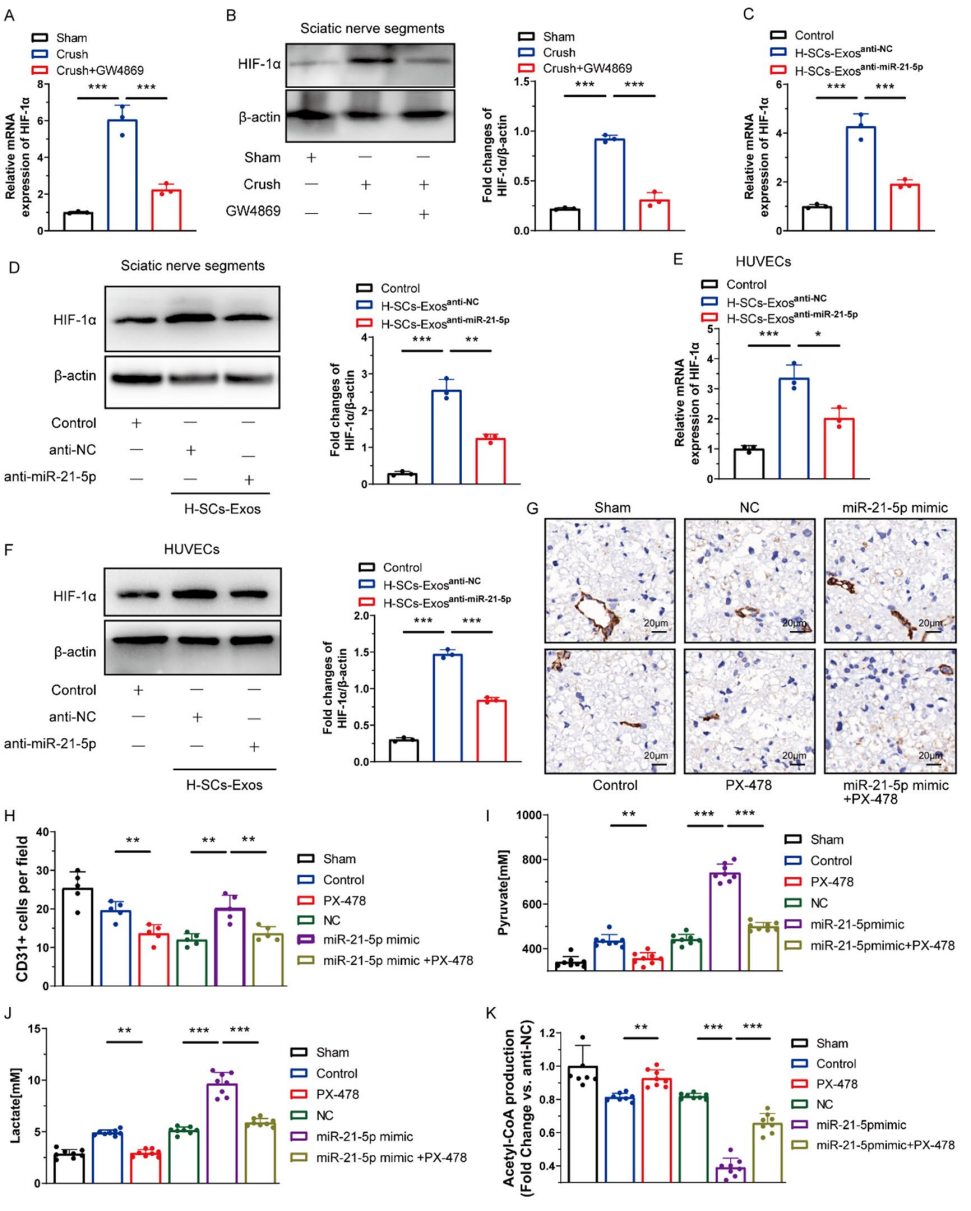
H-SCs-Exos-derived miR-21-5p stabilizes HIF-1α to enhance endothelial glycolysis-mediated intraneural revascularization. (**A**). qRT-PCR assay for expression of HIF-1α mRNA at injured sciatic nerve sites after inhibiting exosomal shuttle with GW4869 administration (*n* = 3 rats/group). (**B**). Western blot assay for HIF-1α protein expression after GW4869 administration (*n* = 3 rats/group). (**C, D**). HIF-1α mRNA and protein expression at injured sciatic nerve sites after saline, H-SCs-Exos^anti−NC^, or H-SCs-Exos^anti−miR−21−5p^ administration (*n* = 3 rats/group). (**E, F**). HIF-1α mRNA and protein expression of HUVECs after PBS, H-SCs-Exos^anti−NC^, or H-SCs-Exos^anti−miR−21−5p^ administration (*n* = 3 rats/group. (**G, H**). Representative immunohistochemical staining of CD31 (brown) for evaluating intraneural revascularization after miR-21-5p mimic and PX-478 treatment (DAPI, blue; *n* = 5 rats/group). (**I–K**). Pyruvate, lactate, and acetyl-CoA production at injured sciatic nerve sites after miR-21-5p mimic and PX-478 administration (*n* = 8 rats/group). ^*^P < 0.05, ^**^P < 0.01, ^***^P < 0.001. Bars represent group means ± SD. A Student’s *t* test was for comparisons. LDHA: Lactate dehydrogenase A; PDH-E1α: Pyruvate dehydrogenase E1 alpha

Hypoxia-induced factors alpha (HIF-1α), a transcriptional factor responsible for mediating glycolysis in the hypoxic microenvironment of the injury site [34, 35], was assayed in injury-site sciatic nerve segments and HUVECs at the transcriptional and translational level. The findings revealed that the mRNA and protein expression of HIF-1α significantly increased after SCI and was counteracted by the local injection of GW4869 (Fig. 6A and B). When the sciatic nerve was locally treated with saline (control), H-SCs-Exos^anti-NC^, or H-SCs-Exos^anti-miR-21−5p^ after crush injury, the results showed that H-SCs-Exos^anti-NC^ administration enhanced the mRNA and protein expression of HIF-1α compared to the control group and that H-SCs-Exos^anti-miR-21−5p^ administration counteracted the increase of HIF-1α (Fig. 6C and D).

A higher HIF-1α expression in HUVECs treated with H-SCs-Exos^anti-NC^ than PBS was also observed in vitro that was counteracted by H-SCs-Exos^anti-miR-21−5p^ administration (Fig. 6E and F). To determine whether miR-21-5p regulated HIF-1α-mediated energy metabolism to facilitate post-injury intra-neuro-revascularization, miR-21-5p mimic or inhibitor was transfected to induce overexpression or knockdown of miR-21-5p, respectively, in vivo and in vitro, which was confirmed by quantitative reverse transcription polymerase chain reaction (qRT-PCR; Fig. S6A and S6B). When PX-478, a HIF-1α inhibitor that can cross the neurovascular barrier, was administered to inactivate HIF-1α activity in the injured sciatic nerve, PX-478 significantly inhibited the CD31 + revascularization of the post-injury sciatic nerve and diminished the pro-intra-neuro-revascularization effects of miR-21-5p (Fig. 6G and H).

Metabolic level measurements showed that PX-478 administration significantly abated pyruvate (Fig. 6I) and lactate (Fig. 6J) production and increased acetyl-coA (Fig. 6K) production. Overexpression of miR-21-5p induced the opposite of these actions. Interestingly, PX-478 partially reversed the effects of miR-21-5p. In vitro, the presence of HUVECs with miR-21-5p and PX-478 increased pyruvate (Fig. S7A) and lactate (Fig. S7B) production but decreased acetyl-coA (Fig. S7C) production of HUVECs. Likewise, miR-21-5p overexpression enhanced the ECAR (Fig. S7D) but not the OCR of HUVECs (Fig. S7E), resulting in increased ECAR/OCR ratio (Fig. S7F), whereas PX-478 partially reversed these effects of miR-21-5p on HUVECs. All these data suggest that miR-21-5p promotes the tendency of EC energy metabolism toward glycolysis through enhanced HIF-1α signaling to facilitate intra-neuro-revascularization after PNI.

**Fig. 7:**
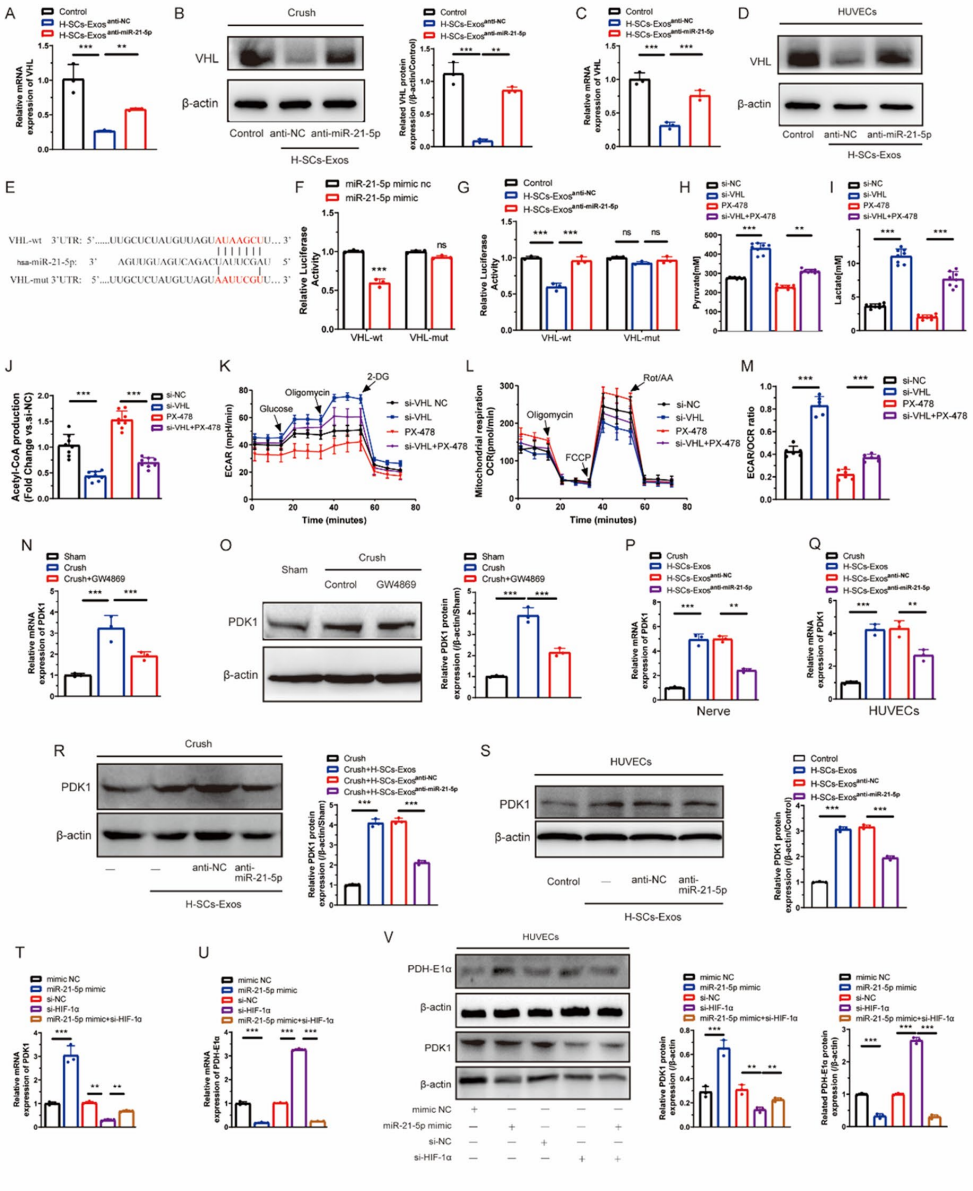
H-SCs-Exos-derived miR-21-5p targets VHL, increasing HIF-1α stabilization to directly or indirectly enhance endothelial glycolysis. (**A**, **B**). VHL mRNA and protein expression at injured sciatic nerve sites after saline (control), H-SCs-Exos^anti−NC^, or H-SCs-Exos^anti−miR−21−5p^ administration (*n* = 3 rats/group). (**C**, **D**). VHL mRNA expression and protein expression in HUVECs after PBS (control), H-SCs-Exos^anti−NC^, or H-SCs-Exos^anti−miR−21−5p^ administration (*n* = 3 rats/group). (**E**). Predicted potential binding sites for miR-21-5p on 3’-UTR of PDH-E1α. (**F**). Dual luciferase activity of HUVECs transfected with VHL-3’UTR luciferase constructs with miR-21-5p mimic or negative control (*n* = 3 rats/group). (**G**). Dual luciferase activity of HUVECs transfected with VHL-3’UTR luciferase constructs with saline (control), H-SCs-Exos^anti−NC^, or H-SCs-Exos^anti−miR−21−5p^ (*n* = 3 rats/group). (**H–J**). Pyruvate, lactate, and acetyl-CoA production of HUVECs transfected with si-NC or si-VHL and with or without PX-478 (*n* = 6 rats/group). (**K**). HUVEC ECAR assay for evaluating cellular glycolytic level (*n* = 6 rats/group). (**L**). HUVEC OCR assay for evaluating cellular mitochondrial respiration (*n* = 6 rats/group). (**M**). ECAR/OCR ratio calculation for further analysis. (**N**, **O**). PDK1 mRNA and protein expression of post-injury sciatic nerves with or without GW4869 administration (*n* = 3 rats/group). (**P**, **Q**). PDK1 mRNA expression in vivo and vitro after treatment with saline or PBS (control), H-SCs-Exos, H-SCs-Exos^anti−NC^, or H-SCs-Exos^anti−miR−21−5p^ (*n* = 3 rats/group). (**R**, **S**). PDK1 protein expression in vivo and in vitro after treatment with saline or PBS (control), H-SCs-Exos, H-SCs-Exos^anti−NC^, or H-SCs-Exos^anti−miR−21−5p^ (*n* = 3 rats/group). (**T–V**). mRNA and protein expression of PDK1 and PDH-E1α in HUVECs with or without miR-21-5p mimic and si-HIF-1α transfection (*n* = 3 rats/group). ^*^P < 0.05, ^**^P < 0.01, ^***^P < 0.001. Bars represent group means ± SD. A Student’s *t* test was used for comparisons. HUVECs: Human umbilical vein endothelial cells; VHL: von Hippel-Lindau; ECAR: Extracellular acidification rate; OCR: Oxygen consumption rate; LDHA: Lactate dehydrogenase A; PDH-E1α: Pyruvate dehydrogenase E1 alpha; PDK1: Pyruvate dehydrogenase kinase 1

To further determine the downstream targets of miR-21-5p, we employed TargetScan, miRanda, and miRTarbase, three independent online bioinformatics databases. miR-21-5p targets contributed to HIF-1α activity prediction focused on VHL, an upstream gene mediating the post-translationally ubiquitinated degradation of HIF-1α. H-SCs-Exos^anti-NC^ administration further aggravated the injury-stimulated downregulation of VHL mRNA and protein, while H-SCs-Exos^anti-miR-21−5p^ administration reversed this downregulation (Fig. 7A and B). In vitro, the protein and mRNA expression of VHL was lower in HUVECs co-incubated with H-SCs-Exos^anti-NC^, whereas H-SCs-Exos^anti-miR-21−5p^ administration partly rescued H-SCs-Exos^anti-NC^-decreased VHL expression (Fig. 7C and D).

If miR-21-5p directly binds to the predicted target region of the VHL mRNA, it could be confirmed by dual-luciferase report assays via co-transfecting HUVECs with a plasmid containing wild- or mutant-type VHL 3’UTR and miR-21-5p mimic or mimic NC. The predicted potential binding sites for miR-21-5p would be on the 3’-UTR of PDH-E1α (Fig. 7E). There was lower luciferase activity in the co-transfected wild-type and mimic group as well as no appreciable change in the mutated group (Fig. 7F). Consistently, H-SCs-Exos decreased the luciferase activity of pVHL 3’UTR but not that of mutant pVHL 3’UTR (Fig. 7G). Interestingly, H-SCs-Exosanti-miR-21-5p could not inhibit luciferase activity (Fig. 7G).

Knock-down of VHL of HUVECs with si-VHL revealed that HIF-1α expression was strongly upregulated at the transcriptional and translational levels (Fig. S8A and S8B). Metabolite detection revealed that VHL depletion with si-VHL increased pyruvate (Fig. 7H) and lactate (Fig. 7I) production and decreased acetyl-coA (Fig. 7J) production. Unsurprisingly, PX-478 reversed these phenomena (Fig. 7H and J). VHL depletion enhanced the ECAR (Fig. 7K) and inhibited the OCR (Fig. 7L) of HUVECs, increasing the ECAR/OCR ratio, while PX-478 diminished these effects. These data indicate that exosomal miR-21-5p enhances HIF-1α-mediated endothelial glycolysis by targeting VHL.

Previous studies demonstrated that HIF-1α inhibits the mitochondrial tricarboxylic acid (TCA) cycle by activating pyruvate dehydrogenase kinase-1 (PDK1) to suppress PDH-E1α [36–40]. In this study, we observed upregulated PDK1 in mRNA and increased protein levels after PNI, which was counteracted by GW4869 administration (Fig. 7N and O). Interestingly, H-SCs-Exos^anti-NC^ administration further enhanced PDK1 upregulation but was counteracted by depletion of miR-21-5p (H-SCs-Exos^anti-miR-21−5p^) *i*n vivo and vitro (Fig. 7P–S). To determine whether exosomal miR-21-5p-increased HIF-1α accumulation led to PDK1 activation inhibiting endothelial OXPHOS, we established a co-transfected system with miR-21-5p NC/mimic and si-NC/HIF-1α. The expression of miR-21-5p (Fig. S6B) and HIF-1α (Fig. S8C–D) was successfully overexpressed and knocked down, respectively.

**Fig. 8:**
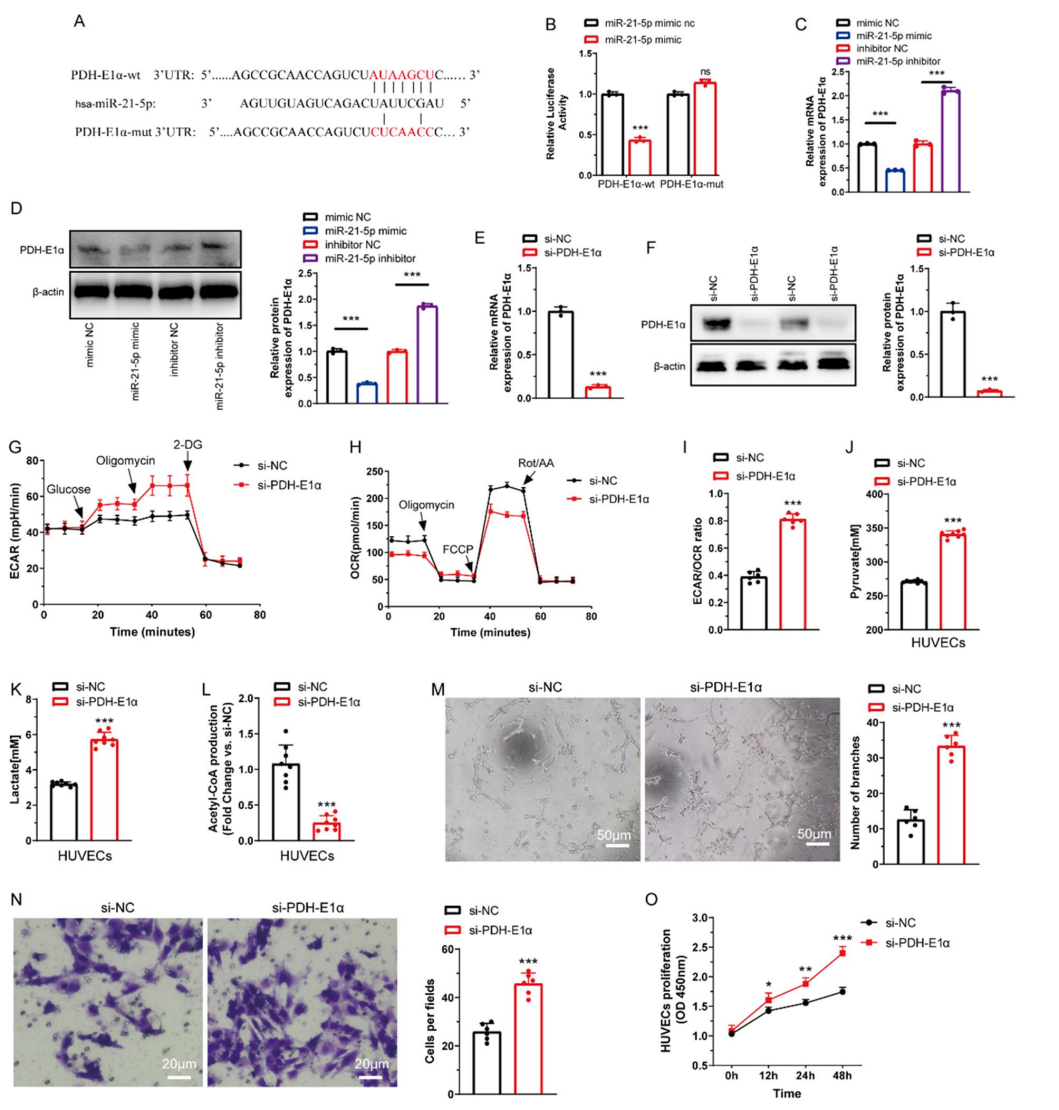
miR-21-5p targets PDH-E1α to inhibit mitochondrial respiration-related OXPHOS of HUVECs. (**A**). Predicted potential binding sites for miR-21-5p on 3’-UTR of PDH-E1α. (**B**). Dual-luciferase reporter assay confirming PDH-E1α is a target gene of miR-21-5p (*n* = 3 rats/group). (**C**). qRT-PCR assay showing PDH-E1α mRNA expression in HUVECs after transfection of miR-21-5p mimic and inhibitor (*n* = 3 rats/group). (**D**). Western blot showing PDH-E1α protein expression level after transfection of miR-21-5p mimic and inhibitor (*n* = 3 rats/group). (**E**, **F**). mRNA and protein expression of PDH-E1α in HUVECs after knockdown of PDH-E1α with si-PDHE1α (*n* = 3 rats/group). (**G**). ECAR, (**H**). OCR, and (**I**). ECAR/OCR ratio of HUVECs after transfection with si-PDHE1α. (**J**–**L**). Pyruvate, lactate, and acetyl-CoA production of HUVECs after transfection with si-PDHE1α (*n* = 8 rats/group). (**M**). Tube-formation capacity assay of HUVECs after transfection with si-PDHE1α (*n* = 6 rats/group). (**N**). Transwell assay for invasion capacity of HUVECs after transfection with si-PDHE1α (*n* = 6 rats/group). (**O**). CCK-8 assay for proliferation capacity of HUVECs after transfection with si-PDHE1α (*n* = 6 rats/group). ^*^P < 0.05, ^**^P < 0.01, ^***^P < 0.001. Bars represent group means ± SD. Student’s *t* test was used for comparisons. HUVECs: Human umbilical vein endothelial cells; OXPHOS: Oxidative phosphorylation; ECAR: Extracellular acidification rate; OCR: Oxygen consumption rate; PDH-E1α: Pyruvate dehydrogenase E1 alpha; PDK1: Pyruvate dehydrogenase kinase 1; CCK-8: Cell counting kit-8

Surprisingly, further results at the transcriptional translation level revealed a remarkable increase in PDK1 and a decrease in PDH-E1α after miR-21-5p mimic treatment, with the knockdown of HIF-1α with si-HIF-1α counteracting PDK1 increase but not rescuing PDH-E1α expression (Fig. 7T–V), indicating another process may be significantly inhibiting PDH-E1α by miR-21-5p.

In addition, we reviewed the targets of miR-21-5p in the TargetScan and miRanda bioinformatic database and found that PDH-E1α with a conserved binding site for miR-21-5p might be a responsible downstream target (Fig. 8A). If miR-21-5p directly binds to the predicted target region of the PDH-E1α mRNA, it would be confirmed by dual-luciferase report assays showing co-transfection of HUVECs with a plasmid containing wild- or mutant-type PDH-E1α 3’UTR and miR-21-5p mimic (Fig. 8A). There was low luciferase activity in the wild-type group transfected with miR-21-5p mimic and no appreciable change in the mutated group (Fig. 8B). qRT-PCR and western blot assay showed that miR-21-5p mimic inhibited the expression of PDH-E1α, and inhibitor increased that (Fig. 8C and D). Our data suggest that the direct inhibition of PDH-E1α is likely the main manner in which miR-21-5p inhibits endothelial OXPHOS.

Previous studies have elucidated that inhibition of PDH-E1α could variously enhance lactate-production glycolysis [41–43]. In the present study, PDH-E1α in HUVECs was effectively knocked down via transfecting siRNA (si-PDH-E1α), as shown in Fig. 8E and F. Downregulation of PDH-E1α enhanced the ECAR (Fig. 8G) and weakened the OCR (Fig. 8H) of HUVECs, increasing the ECAR/OCR ratio (Fig. 8I); simultaneously, metabolite assays showed an increase in pyruvate (Fig. 8J) and lactate (Fig. 8K) production but a decrease in acetyl-CoA (Fig. 8L) production. Pro-angiogenic potentiality assay showed that knockdown of PDH-E1α promoted tube-formation capacity (Fig. 8M), invasion capacity (Fig. 8N), and proliferation (Fig. 8O). These data suggest that exosomal miR-21-5p-mediated PDH-E1α inhibition weakens OXPHOS and variously strengthens the glycolysis of intraneural endothelial cells, promoting intraneural revascularization synchronously after PNI.

In summary, our findings indicate that H-SC-Exo-augmented miR-21-5p transition facilitates intraneural endothelium-initiated revascularization by targeting VHL to enhance HIF-1α-accumulation-mediated glycolysis and targeting PDH-E1α to weaken mitochondrial OXPHOS.

## Discussion

This study demonstrated that SCs-Exos enhance glycolytic metabolism and synchronously repress endothelial OXPHOS, facilitating intraneural revascularization, axonal regeneration, and functional recovery after PNI. Transferred miR-21-5p was primarily responsible for this metabolic shift via targeting VHL to enhance HIF-1α stabilization-mediated endothelial glycolysis and undermine PDH-E1α-mediated OXPHOS. Under hypoxic conditions, the miR-21-5p quantity significantly increased, which further augmented the metabolic shift in favor of glycolysis. Concurrently, the two pathways reciprocally affected each other and conjointly enhanced endothelial glycolysis. Together, these data reveal a novel and comprehensive metabolic means of facilitating axon regeneration and functional recovery from PNI.

It is well-accepted that INVS development and reconstruction are strongly connected with neurogenesis and nerve regeneration in PNS. Both the promotion of INRV and the induction of adequate vascularization of artificial nerve grafts significantly enhance pro-neural regenerative effects [10, 44–47]. However, little is known about the specific role and underlying mechanisms of INV in PNI. Vascularization in PNS is divided into two longitudinal systems: the extra-neural vascular system (ENVS), located peri-fascicularly for connection to external vascular systems by branches, and the INVS, located intra-fascicularly for direct contact with neural cells. In this study, we mainly focused on the reconstruction of INVS after PNI. The complex INVS needed at least 7 days for reconstruction and 14 days for sufficient pre-vascularization in vitro but only 3 to 5 days for sufficient polarized blood vessel formation in vivo [9, 10, 12, 45]. The activated regenerative program of neural cells and non-neural cells as well as enhanced intercellular communication under different pathological and physiological conditions, particularly in ischemic and hypoxic microenvironments, is crucial for post-injury nerve regeneration of PNS [7, 12, 48–52], which may explain the faster INRV in vivo and more significant pro-INRV effects of H-SCs-Exos that we observed.

In this study, we found that the INRV process after PNI was significantly disrupted by GW4869, an inhibitor of the exosomal shuttle. Emerging evidence demonstrates that SCs-Exos play a crucial role in nerve development and post-injury regeneration after PNI [32, 53–55]. Myelinated SCs negatively regulate intraneural angiogenesis during the postnatal period, significantly decreasing intraneural vessel density [56]. However, SCs and their derivatives, such as netrin-1, also have prominent pro-angiogenic potential in vitro and in vivo [56–58]. There is controversy regarding the effects of SCs on the INVS of PNS. Hypoxic precondition could enhance the pro-angiogenic and pro-neural regenerative effects of cells and their exosomes [59–61]. Similarly, in the central nervous system under ischemic-hypoxic conditions, the interactions between oligodendrocytes and endothelial cells promote neoangiogenesis to attenuate brain injury [62, 63]. Thus, we speculate that SCs are responsible for maintaining the homeostasis of INVS density and the ischemic-hypoxic conditions caused by disruption of INVS or mechanical stimuli after PNI, which induce the dedifferentiation of SCs and shifts the effects of SCs on the INVS by limiting their promotion.

Hypoxic or injurious stimuli are also intensive activators of glycolysis. The manipulation of EC glycolytic activity enabled us to accelerate or delay angiogenesis and organic regeneration [15, 17, 51, 64–69]. Several studies have reported that enhancement of OXPHOS inhibits angiogenic sprouts and extensions, whereas inhibition of OXPHOS promotes them [69–72]. Nerves could switch on EC metabolism in favor of glycolysis via releasing adrenergic and cholinergic neurotransmitters to promote angiogenesis [71, 73, 74]. In PNS, the upregulated glycolytic activity of SCs delays the degeneration of perturbed axons after PNI [75].

Several studies have found that exosomes are intimately implicated in intercellular metabolic regulation [76–79]. In the present study, we determined that SCs are also involved in the regulation of EC metabolic activities in favor of glycolysis through intercellular communication mediated by exosomes, whereas neurotransmitters promote post-injury INRV of PNS. MicroRNAs, a group of small non-coding RNAs with 18 to 25 nucleotides that are the most studied contents in the exosome, participate in various physio-pathological processes, such as hematopoiesis, aging, and tissue repair [80, 81]. miR-21-5p, a hypoxia-responsive and pro-angiogenic miRNA, was found to be highly enriched in SCs and their exosomes in our previous study and other studies [26, 31, 32]. In the present study, we consistently found strong upregulation of miR-21-5p in hypoxic-precondition SCs and their exosomes as well as injured nerve segments.

Recent studies have reported that miR-21-5p could also enhance glycolysis and concurrently attenuate OXPHOS in other cell types [40, 82, 83]. In this study, we determined that miR-21-5p is also a primary molecule of SCs-Exos in regulating EC metabolism and revascularization capacity to facilitate INRV and nerve regeneration after PNI. HIF-1α, a key transcription factor that regulates the expression of multiple downstream genes in a hypoxic environment after tissue injury, has been demonstrated to be extensively involved in glycolytic metabolism [84, 85]. Previous studies have reported that deletion of VHL, a component of the E3 ubiquitin ligase complex that binds to HIF-1α and leads to its polyubiquitination of the α subunit and degradation by the proteasome under normoxic conditions, strengthens HIF-1α stabilization and accumulation to promote glycolysis and angiogenesis [35, 86, 87].

Although it is well accepted that HIF-1α is a key promoter of nerve regeneration and neoangiogenesis [50, 61, 75, 88–92], the effects of HIF-1α on INRV after PNI remain unclear. In this study, we identified a new role of HIF-1α in regulating EC metabolism and vascularization capacity after PNI, as well as determined that SC-Exo-derived miR-21-5p directly targets VHL to regulate HIF-1α accumulation-mediated glycolysis of ECs to facilitate INRV after PNI. HIF-1α accumulation could simultaneously inhibit mitochondrial OXPHOS via the PDK1/PDH-E1α pathway [36–39]. However, we observed that the knockdown of HIF-1α with si-HIF-1α slightly reversed the inhibition of miR-21-5p mimic on endothelial OXPHOS. In further investigation, we found that PDH-E1α, the key enzyme catalyzing the conversion of pyruvate to acetyl-CoA for mitochondrial OXPHOS, is also a direct target of miR-21-5p in EC and that inhibition of PDH-E1α-controlled OXPHOS complementarily enhances the glycolysis of ECs after PNI, as previous studies reported [40, 93, 94].

This study had several limitations that should be considered when reviewing the findings. First, we were unable to elucidate the effects of other molecules, such as mRNA, proteins, and other non-coding RNAs in hypoxic-preconditioned SC-Exos in EC metabolism and vascularization capacity. Second, we did not explore other potential targets of miR-21-5p in regulating EC function after PNI. Third, we explored only the effects and underlying mechanisms of miR-21-5p from SC-Exos on INRV and not those of other sources. Despite these limitations, our study identified a novel role of SCs in facilitating INRV via the exosomal shuttle; a mechanism by which miR-21-5p regulates EC energy metabolism in favor of glycolysis; and a crucial link among exosomes, metabolism, angiogenesis, and nerve repair after PNI.

## Conclusion

In this study, it was demonstrated that SC-Exo-regulated metabolic alteration of ECs via transferring of miR-21-5p and simultaneously targeting VHL to enhance HIF-1α accumulation-mediated glycolysis and PDH-E1α to inhibit mitochondrial OXPHOS, facilitated post-injury INRV. In addition, hypoxic precondition strongly increased miR-21-5p expression to further skew energic metabolism in favor of glycolysis after PNI. Our findings revealed a novel intrinsic mechanism of INRV after PNI, the targeting of which may provide a promising therapeutic strategy for post-injury regeneration and repair of PNS.

## Materials and methods

### Animals and sciatic nerve crush injury model

We conducted all experiments on animals in this study following the guidelines of the Guide for the Care and Use of Laboratory Animals of the US National Institutes of Health. The Institutional Review Board and Ethics Committee of the Third Affiliated Hospital of Sun Yat-Sen University approved all work in this study. We obtained adult male Sprague–Dawley rats weighing 150 to 200 g and aged 6 to 8 weeks from the Animal Experiment Center, North Campus of Sun Yat-sen University. We housed the animals in a humid room at a constant temperature of 26 °C on a 12-h light-and-dark cycle and fed them with a standard chow preparation and water ad libitum. We housed the rats in an experimental environment for seven days to adapt them before the surgical procedure.

We performed the surgical procedure of sciatic nerve crush injury according to a previous study [95]. In brief, we anesthetized all experimental rats with isoflurane and aseptically operated on a sterile operative table after thorough disinfection. We made a 1.5-cm longitudinal incision on the dorsal side of the lower right limb and bluntly separated the subcutaneous fascia and muscles to expose the upper segment of the sciatic nerve. We used the same medium-sized straight hemostatic forceps to press the upper sites of the sciatic nerve 5 mm at the lower edge of the piriformis muscle to the last grid with an approximately 54 N compressive force with the tip for 30 s until the nerve became transparent. After rats being treated with saline, GW4869, Nor-SCs-Exos, and Hypoxia-SCs-Exos, we sutured the skin layer by layer. We intraperitoneally administered GW4869 (2.0 µg/g body weight) daily from five days before the procedure to five days after to inhibit the intraneural exosome paracrine.

### Purification and culture of primary SCs

Primary SCs were obtained from the sciatic nerves of neonatal rat pups according to a previous study [96, 97]. In brief, the collected sciatic nerve segments were sliced and digested in 5 ml of 0.1% II collagenase solution and 5 ml of 0.25% trypsin solution for 45 min at 37 °C and 5% CO2. The supernatants were removed after centrifugation for 5 min at 400 g. The pellet was digested in 5 ml of 0.1 IV collagenase solution for 30 min and neutralized by Dulbecco’s Modified Eagle’s medium (DMEM) containing 15% fetal bovine serum (FBS). The cell suspension was filtered through a 40-µm strainer and centrifuged for 5 min at 400 g before supernatant removal. The washed cell pellet with DMEM containing 15% FBS, 100 µg/mL of streptomycin, and 100 U/mL of penicillin was centrifuged again at 400 g for 5 min followed by supernatant discard. Finally, resuspended cells with DMEM D-valine containing 10% FBS, 2 mM of L-glutamine, 20 µg/mL of bovine pituitary extracts, 100 µg/mL of streptomycin, 100 U/mL of penicillin, 5 µM of forskolin, and 0.25 µg/mL of amphotericin B were plated on a 3-mm petri dish precoated with 0.01% poly-l-lysine and 1 µg/mL of laminin for 24 h in an incubator at 37 °C and 5% CO2 and incubated in same culture condition. The medium was changed every two days until 85% confluency was achieved. Passages 3 to 7 (P3–P7) of primary SCs were used for further experimentation.

### Extraction and identification of exosomes

SC-Exos were purified from the supernatant through ultracentrifugation according to previous studies [28, 29]. Briefly, the cultured supernatant was collected and centrifuged at 300 ×*g* for 10 min and then at 2000 ×*g* for 20 min to remove cells and debris. The collected supernatant was transferred to a new tube for further centrifuging at 10,000 ×*g* for 30 min using a 0.22-µm filter to remove all cellular debris. The collected supernatant was sequentially ultracentrifuged twice at 100,000 ×*g* for 70 min (Beckman Coulter Optima L-100 XP Ultracentrifuge, SW 32 Rotor; Beckman Coulter Indianapolis, IN, USA). All centrifugation procedures were performed at 4 °C. The pellets were resuspended and stored at − 80 °C and the exosomal morphology characterized by TEM at 100 kV (JEM-1200EX; Tokyo, Japan) and the concentrations and diameters of SC-Exos measured with a Nanosight Tracking Analyzer (NS300; Malvern, UK). Finally, a western blot was performed to identify the exosome-related factors. The primary antibodies were listed in Table S3 including anti-CD63, anti-CD9, anti-TSG101 and anti-calnexin.

### Exosome uptake assay

Purified normoxic and hypoxic SCs-derived exosomes (Nor-SCs-Exos and Hypo-SCs-Exos) were labeled for 1 h with 1 µM of DiR staining reagents (Umibio, Shanghai, China) in a 37 °C incubator according to the manufacturer’s instructions as previously reported with slight modifications [98]. DiR-labeled Nor-SCs-Exos and Hypo-SCs-Exos were co-cultured with HUVECs at a concentration of 100 µg/ml in a 37 °C-cell incubator with 5% CO2 for 24 h. The nuclei were counterstained with DAPI for 15 min at room temperature. Images were taken with a confocal fluorescence microscope (ZEISS, Jena, Germany).

### Cell transfection and treatment

HUVECs were plated in a 6-well plate and transfected with 30 nM of miR-21-5p mimic NC, miR-21-5p mimic, miR-21-5p inhibitor (RiboBio, Guangzhou, China), 50 nM of si-NC or si-VHL, si-NC or si-HIF-1α, and si-NC or si-PDH-E1α (RiboBio) when cells reached 50 to 60% confluence. The sequences of the small interfering RNAs (siRNAs), miR-21-5p mimic, and inhibitors used in this study are shown in Table S1. siRNA Lipofectamine 3000 (Thermofisher Scientific, Agawam, MA, USA) was used for cell transfection experiments according to the manufacturer’s instructions. HUVECs were treated with 200 µg/ml of N-SCs-Exos or H-SCs-Exos.

### Dual-luciferase reporter gene assay

Luciferase reporter gene assay constructs (3′UTR-NC, 3′UTR-VHL, 3′UTR-VHL-mutant, 3′UTR-PDH-E1α, and 3′UTR- PDH-E1α-mutant), miRNA (miRNA-NC or miR-21-5p), and Renilla luciferase (GeneChem, Shanghai, China) were co-transfected into HUVECs cells (Shanghai Institute of Cell Research, Chinese Academy of Sciences) using Lipofectamine 3000 (Thermo Fisher Scientific). After transfection for 48 h, cells were harvested and lysed. A Dual-Luciferase Reporter Assay System Kit (Promega, Madison, WI, USA) was used to measure luciferase activity according to the manufacturer’s instructions. Each experiment was repeated three times.

### Tube formation assay

Transfected or treated HUVECs were resuspended with DMEM containing 10% PBS, 100 µg/mL of streptomycin, and 100 U/mL of penicillin and then added into a 96-well culture plate precoated with Matrigel (BD Biosciences, Franklin Lakes, NJ, USA) at a density of 2 × 10^4^ cells per well for 10 to 12 h at 37 °C. Images of tubule formation was visualized and taken under an inverted fluorescent microscope (Nikon, Tokyo, Japan). Tubes were manually counted and the branches were quantified using Image J software.

### Cell invasion, migration, proliferation assay

HUVEC invasion was assayed using a 24-well Transwells plate. In brief, 600 µl of DMEM containing 10% FBS, 100 µg/mL of streptomycin, and 100 U/mL penicillin was added to the lower chamber as a chemoattractant. Transfected exosome-treated HUVECs at a 1 × 10^5^ density were added to the upper chamber precoated with 200 µl of Matrigel diluted with serum-free DMEM at a ratio of 1:8 for 48 h at 37 °C. Non-invasive HUVECs were removed, and invasive cells were fixed with 100% methyl alcohol for 20 min before being stained with 0.1% crystal violet. Invasive cells were counted and images were taken using a brightfield microscope. HUVEC migration was assessed using scratch assay. Transfected or exosome-treated HUVECs were cultured in a 6-well culture plate until 70% confluence. A 200-µl pipette tip was used to scratch three liner wounds per well, and HUVECs were washed three times with sterile PBS buffer to remove the floating cells. HUVECs were continually cultivated in a 37 °C 5% CO_2_ incubator. Scarifications were recorded one-to-one under an inverted phase-contrast microscope (ECLIPSE Ti2-U; Nikon) at 0 and 48 h after the scratch.

EdU assay was used to evaluate cell proliferation. Briefly, transfected or treated HUVECs at a density of 5 × 10^4^ were seeded onto a 48-well plate. A 10-µM EdU-labeling reagent (Ribobio) was added to the HUVECs and co-incubated with them for 2 h. After being washed with PBS, HUVECs were fixed with 4% paraformaldehyde for 30 min, neutralized by 2 mg/ml of glycine, and permeabilized by 0.5% TritonX-100 for 15 min. HUVEC proliferation was detected using a Cell-Light EdU Apollo567 In Vitro Kit (Ribobio) and images were acquired using a fluorescent microscope (ECLIPSE Ti2-U; Nikon). Cell counting kit-8 (CCK-8) assay was used to evaluate cell viability. Briefly, HUVECs at a density of 1 × 10^4^ were seeded onto a 96-well plate and transfected with the indicated oligonucleotides or treated with indicated exosomes. After 0, 24, 48, and 72 h, 10 µl of CCK-8 reagent was added to HUVECs per well for 2 h, and the absorbance (optical density value) at 450 nm was measured using an Eon Microplate Spectrophotometer (Biotek, Winooski, Vermont, USA).

### Extracellular acid rate and oxygen consumption rate assay

ECAR and OCR were determined using a Seahorse XFe^96^ Extracellular Flux Analyzer (Seahorse Bioscience, North Billerica, MA, USA) according to the manufacturer’s instructions. ECAR reflects glycolysis and OCR reflects cell respiration. Briefly, HUVECs (8,000 cells/well) were seeded into a Seahorse XFe^96^ cell culture plate containing 100 µl of DMEM supplemented with 10% FBS, 100 µg/mL of streptomycin, and 100 U/mL of penicillin and then treated or transfected with related exosomes or oligonucleotides for 48 h. The DMEM was replaced with a non-buffered medium, and the cell culture plate was maintained in a non-CO_2_ incubator for 60 min. For the ECAR assay, 10 mM of glucose, 2 µM of oligomycin (mitochondrial/ATP synthase inhibitor), and 100 mM of 2-deoxyglucose (2-DG) were injected into each well. For the OCR assay, 2 µM of oligomycin, 1 µM of carbonylcyanide-4-trifluoromethoxy-phenylhydrazone (FCCP), and 1.5 µM of rotenone/antimycin A (Rot/AA) were sequentially auto-injected into each experimental well at specific time points.

### Pyruvate, lactate, and acetyl-CoA production assay

Lactate, pyruvate, and acetyl-CoA levels were separately determined using a Lactate Assay Kit (Jiancheng Bioengineering Institute, Nanjing, China), Pyruvate Assay Kit (Jiancheng Bioengineering Institute), and an Acetyl-Coenzyme Assay Kit (Solarbio, Beijing, China) according to the manufacturer’s protocols. All experiments were performed at least three times.

### Quantitative real-time polymerase chain reaction

Total RNA was extracted from HUVECs, exosomes, and tissues using RNAiso Plus (Takara Bio, Tokyo, Japan). The cDNA was synthesized and amplified using a PrimeScrip RT Reagent Kit (Takara) according to the manufacturer’s protocols. qRT-PCR was performed using TB Green Premix Ex Taq II (Takara). The sequences of primers (Ribobio) used in this study are listed in Table S2.

### Western blot

Processed HUVECs or sciatic tissues were lysed by radioimmunoprecipitation assay (RIPA) using a buffer containing 1% protease inhibitor and 1% phenylmethylsulfonyl fluoridepms (Sigma-Aldrich, St. Louis, MO, USA). Protein concentration was detected using a bicinchoninic acid (BCA) protein assay kit (Beyotime Biotechnology, Haimen, China). Pierce LDS Sample Loading Buffer (4X) (Thermo Scientific) was added to the collected supernatant at a ratio of 1:3 and the protein was maintained in a 70 °C-water bath for 10 min. A total of 20 to 30 µg protein per lane was separated using 7.5 to 12.5% sodium dodecyl-sulfate polyacrylamide gel electrophoresis (SDS-PAGE) and transferred to a polyvinylidene fluoride (PVDF) membrane (Beyotime). After blocking with 5% skim milk at room temperature for 60 min, membranes were incubated with the first antibodies as listed in Table S3 at 4 °C overnight. The next day membranes were washed with 1 × Tris-Buffered Saline, 0.1% Tween 20 Detergent (TBST) three times and incubated with secondary antibodies for 1 h at room temperature. The results were visualized using enhanced chemiluminescence (ECL; Beyotime) and quantified by ImageJ software (ImageJ 1.32 J, National Institutes of Health, Bethesda, MD, USA).

### Immunohistochemical staining

In brief, injured sciatic nerve segments were quickly dissected and fixed with 4% paraformaldehyde for 24 h or overnight at 4 °C, rinsed with 0.1 mM PBS solution, and cryoprotected in 10% sucrose in PBS with 0.1% sodium azide for 24 h before sciatic nerves were cut into 14-µm sections. The primary antibodies and staining used were anti-CD31, anti-LDHA and anti-PDH-E1α as listed in Table S3. The secondary antibodies used were Alexa 488/594 goat anti-rat, anti-rabbit, or anti-mouse (Invitrogen). All immunohistochemistry followed an indirect immunostaining protocol where the peroxidase-diaminobenzidine (DAB) reaction was used for immunostaining the sciatic nerves and DAB (brown) plus DAB-NICHEL (blue) for reactions.

### Immunofluorescence

In brief, the crush-injury segment of the sciatic nerve was collected and fully fixed with 4% paraformaldehyde for 24 h at 4 °C. A fixed sciatic nerve was embedded in the paraffin block and then cut into 5-µm sections. After being blocked in 10% bovine serum albumin (BSA) for 1 h at room temperature, sections were incubated with primary antibodies overnight at 4 °C in a blocking buffer. The sections were then incubated with secondary antibodies for 1 h at room temperature in the dark after being washed 3 times in a blocking buffer. Additional primary antibodies used were anti-NF200 (1:50; Cell Signaling Technology, #30,564) and anti-MBP (1:50; Cell Signaling Technology; #78,896). Nuclei were incubated with DAPI (Beyotime, P0131) for 5 min at room temperature. Images were acquired using a confocal fluorescence microscope (ZEISS, Jena, Germany).

### Sciatic function index measurement

The sciatic nerve index (SFI) is a well-characterized index widely used to assess nerve function and regeneration after sciatic nerve injury. SFI was measured one day before axotomy and nerve crush and at 2 and 8 weeks postoperatively. SFI was calculated using three parameters, including total print length (P; from the tip of the third toe to heel), toe spread (TS; from the first to the fifth toe), and intermediate toe spread (IT; from the second to the fourth toe). The calculative formula is as follows:

$$\text{S}\text{F}\text{I}=\frac{\text{P}{\text{L}}_{\text{R}}-\text{P}{\text{L}}_{\text{L}}}{\text{P}{\text{L}}_{\text{L}}}\times (-38.3)+$$

$$\frac{\text{T}{\text{S}}_{\text{R}}-\text{T}{\text{S}}_{\text{L}}}{\text{T}{\text{S}}_{\text{L}}}\times 109+13.3\times \frac{\text{I}\text{T}{\text{S}}_{\text{R}}-\text{I}\text{T}{\text{S}}_{\text{L}}}{\text{I}\text{T}{\text{S}}_{\text{L}}}-8.8$$

where R is the right side and L is the left side.

### Statistical analysis

Statistical analysis was performed using SPSS 22.0 and GraphPad Prism 8.0 software. The data are expressed as the mean ± standard deviation. Comparisons between different experimental conditions were performed using the Student’s paired *t* test and multiple comparisons were performed using analysis of variance. Statistical significance was established at *P* < 0.05.

### Electronic supplementary material

Below is the link to the electronic supplementary material.

Supplementary Material 1

## Data Availability

The data are available from the corresponding author upon reasonable request.
