# Supplementary material for: Enhancing intraneural revascularization following peripheral nerve injury through hypoxic Schwann-cell-derived exosomes: an insight into endothelial glycolysis
Source: J Nanobiotechnology. 2024 May 24;22:283. doi: 10.1186/s12951-024-02536-y (PMC11127458; doi:10.1186/s12951-024-02536-y)
Supplement: Supplementary file 1 — Supplementary Material 1 [file 12951_2024_2536_MOESM1_ESM.docx]

**Extended Data Figures and Legends**


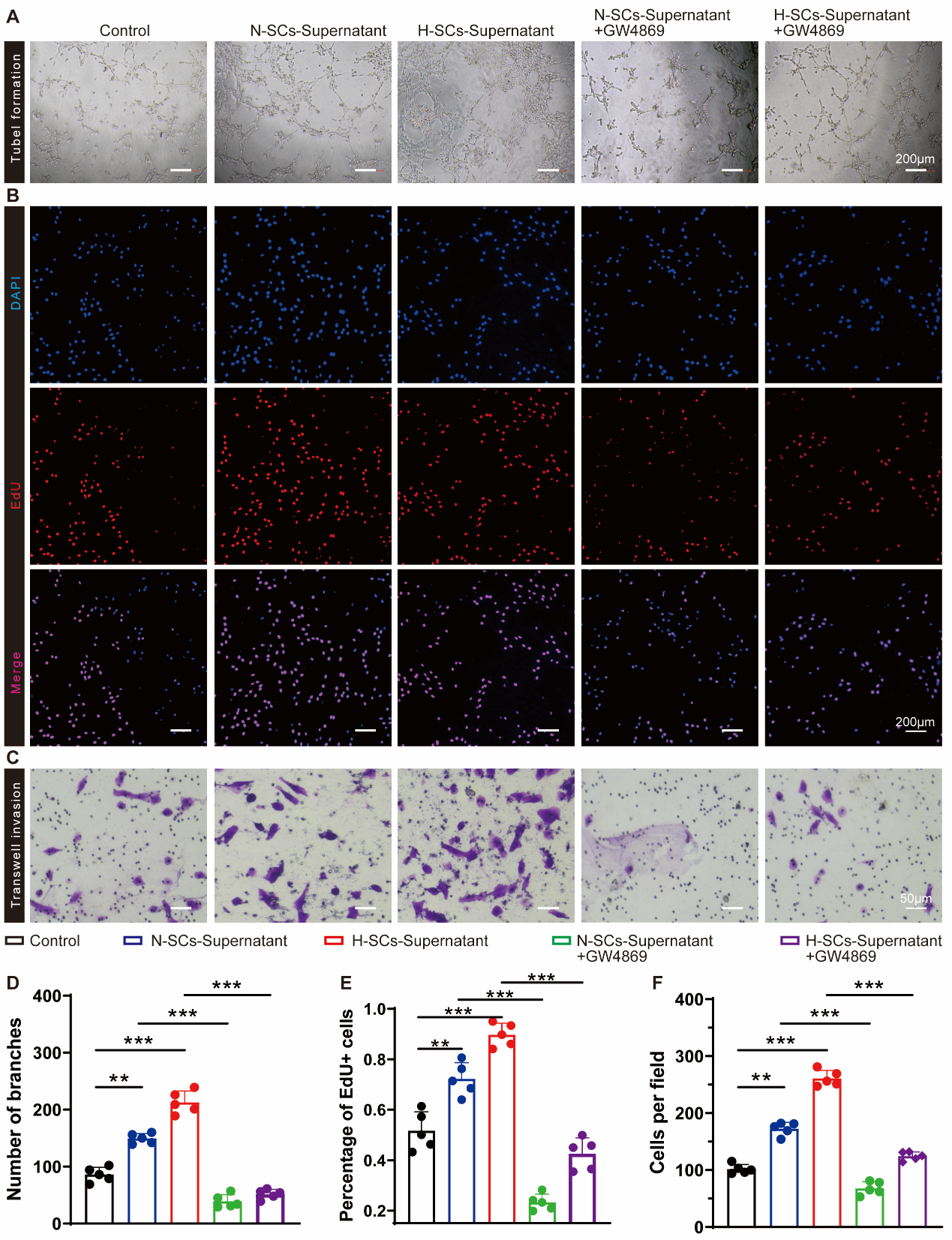


**Figure S1: GW4869 administration for inhibiting exosomal secretion is counteracted by SCs-supernatant-facilitated HUVEC tube formation, proliferation, and invasion capacity.**

**A.** Results of tube-formation capacity assay (scale bar = 200 μm). **B.** Results of EdU assay for proliferation capacity (scale bar = 200 μm). **C.** Transwell assay for invasion capacity of HUVECs after PBS, N-SCs-supernatant, H-SCs-supernatant, N-SCs-supernatant+GW4869, and H-SCs-supernatant+GW4869 treatment (scale bar = 50 μm). **D.** Statistical analysis of HUVEC tube formation shown in (A). **E.** Statistical analysis of HUVEC proliferation shown in (B). **F.** Statistical analysis of HUVEC invasion shown in (C). ^*^*P* < 0.05, ^**^*P* < 0.01, ****P* < 0.001; *n* = 5 rats/group. Bars represent group means ± SD. Student’s *t* test was used for comparisons. SCs: Schwann cells; HUVECs: human umbilical vein endothelial cells.

**
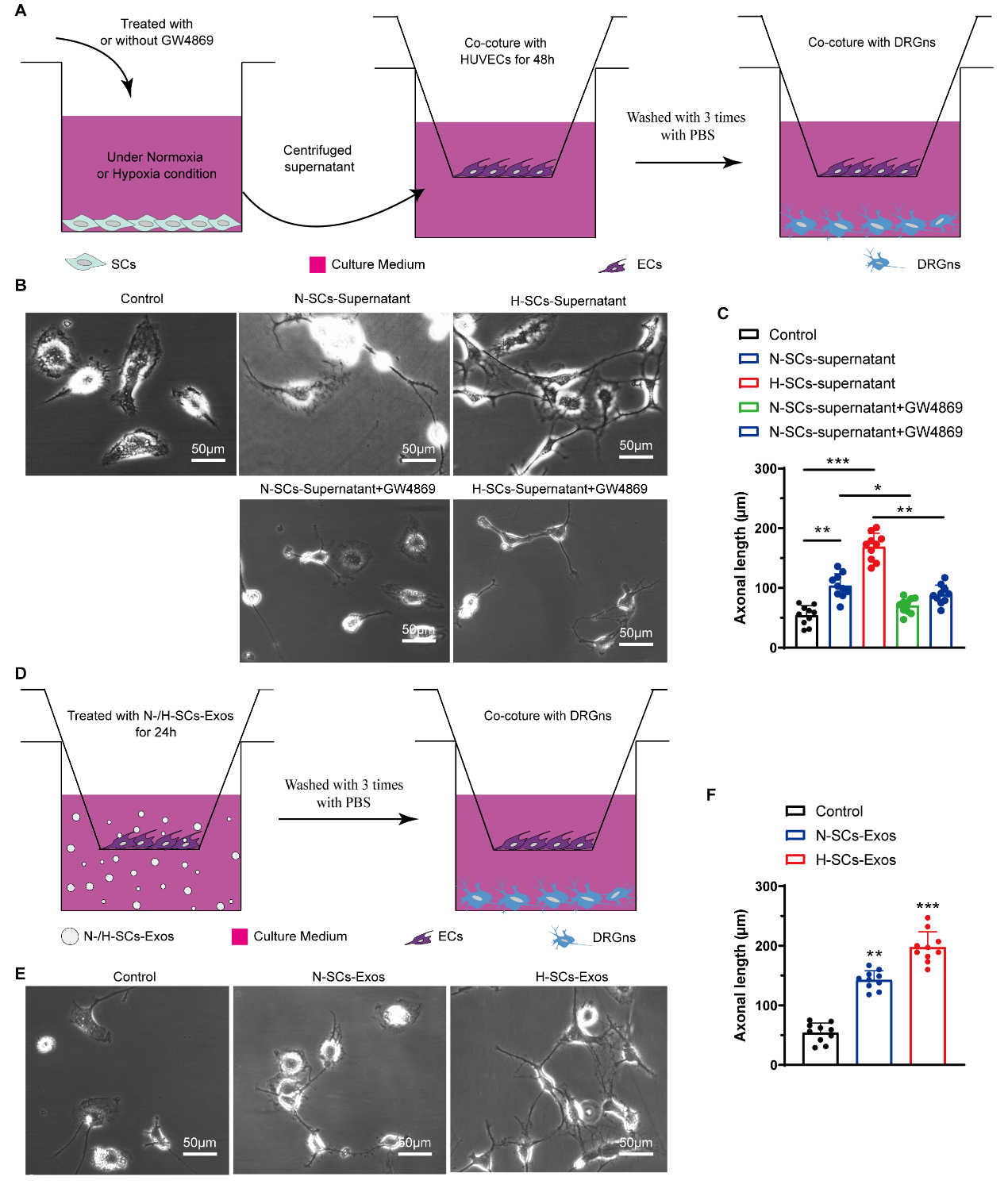
**

**Figure S2: Growth of DRGns after treatment with different condition-derived supernatants with or without GW4869 and exosomes from SCs.**

**A.** Schematic diagram of co-culture system of different conditions of SC-supernatant-treated HUVECs with DRGns. **B, C.** H-SCs-supernatant-treated HUVECs more significantly enhanced the axonal growth of DRGns than H-SCs-Exos-supernatant-treated HUVECs but GW4869 administration counteracted the growth in both conditions (scale bar = 50 μm, *n* = 10 rats/group). **D.** Schematic diagram of the co-culture system of N-/H-SC-Exos-treated HUVECs with DRGns. **E, F.** H-SCs-Exos-treated HUVECs more strongly promoted axonal growth of DRGns than N-SCs-Exos-treated HUVECs (scale bar = 50 μm, *n* =10 rats/group.

^*^*P* < 0.05, ^**^*P* < 0.01, ^***^*P* < 0.001; *n* = 5 rats/group. Bars represent group means ± SD. A Student’s *t* test was used for comparisons. SCs: Schwann cells; HUVECs: human umbilical vein endothelial cells.

**
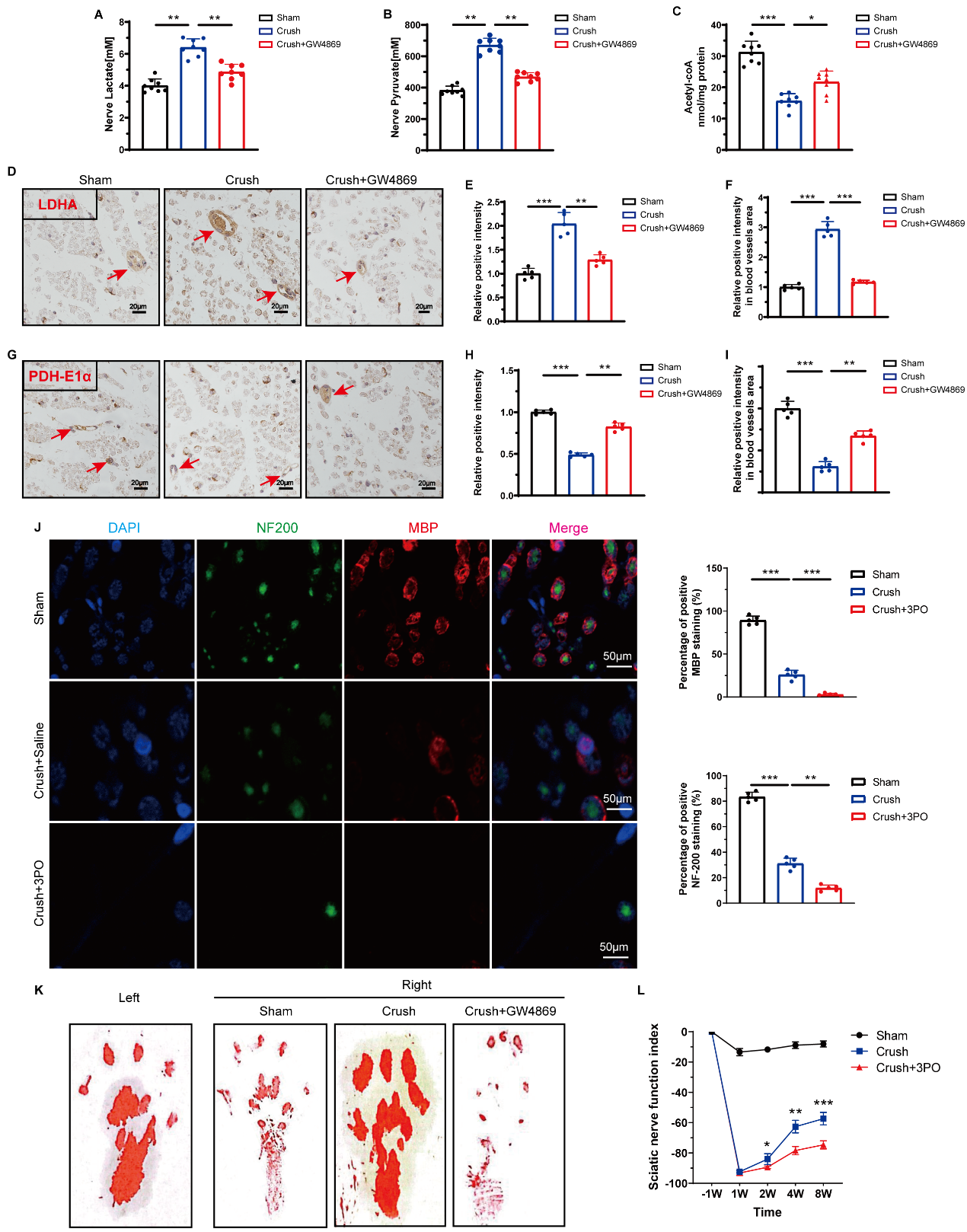
**

**Figure S3: GW4869 administration suppresses injury-induced glycolysis upregulation of intraneural endothelial cells. A.** Lactate production, **B.** pyruvate production, and **C.** acetyl-CoA production assay at injured sciatic nerve with or without GW4869 administration (*n* = 8 rats/group). **D-F.** Representative immunohistochemistry for LDHA expression of entire sciatic nerve or intraneural ECs. The red arrow shows the expression of LDHA at intraneural ECs (scale bar = 20 μm, *n* = 5 rats/group). **G-I.** Representative immunohistochemistry for PDH-E1α expression of the entire sciatic nerve or intraneural ECs. The red arrow shows an expression of PDH-E1α at intraneural ECs (scale bar = 20 μm, *n* = 5 rats/group). ^*^*P* < 0.05, ^**^*P* < 0.01, ^***^*P* < 0.001; *n* = 5 rats/group. Bars represent group means ± SD. A Student’s *t* test was used for comparisons.


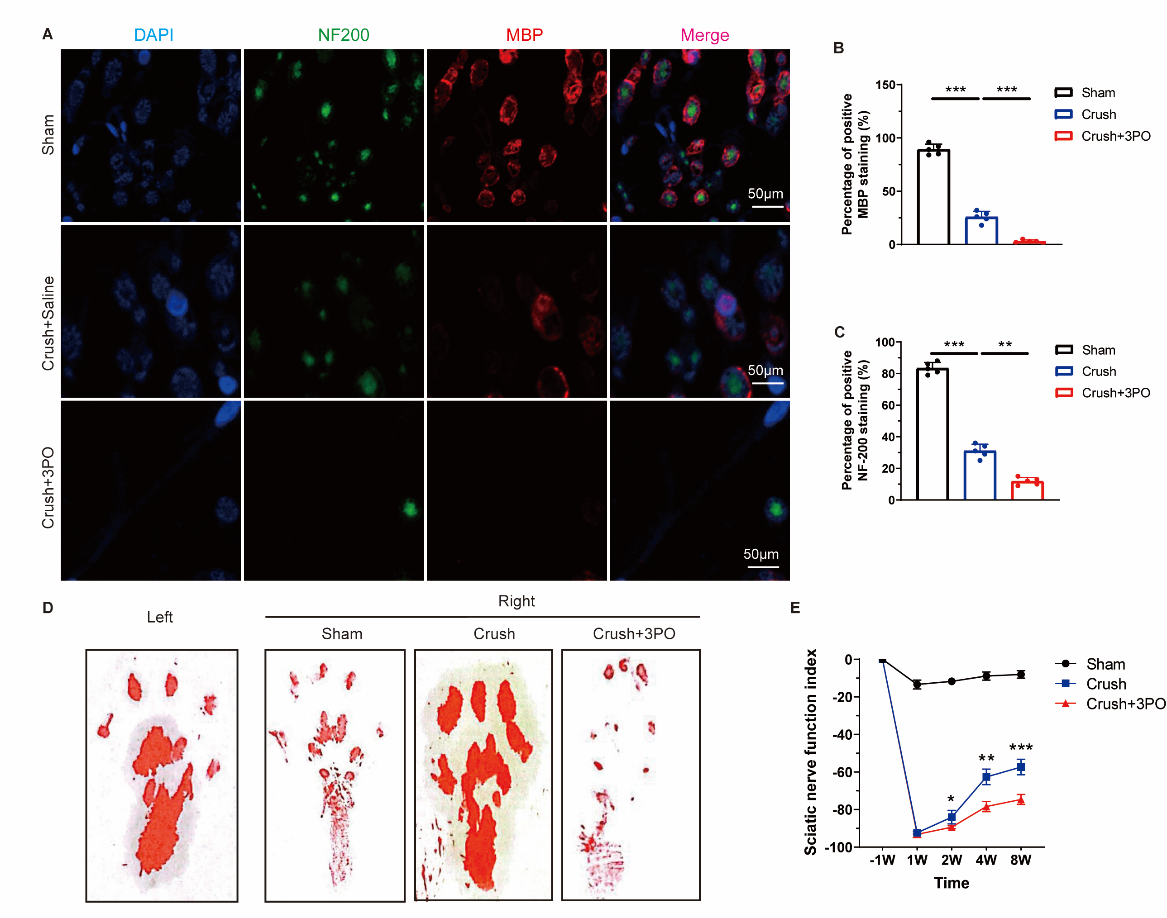


**Figure S4: Glycolysis inhibition by 3PO delays nerve regeneration and functional recovery**

**A.** Representative immunofluorescent staining of neurofilaments (NF-200, green), myeline (MBP, red), and nuclei (DAPI, blue) to evaluate post-injury nerve regeneration (scale bar = 50 μm, *n* = 5 rats/group). **B.** Representative footprints of rats with or without 3PO administration 8 weeks after sciatic nerve crush injury. The left image shows the control group and the right image the sham or operative group. **C.** SFI calculation to evaluate functional recovery from week 1 to 8 after sciatic nerve injury (*n* = 5 rats/group). ^*^*P* < 0.05, ^**^*P* < 0.01, ^***^*P* < 0.001. Bars represent group means ± SD. A Student’s *t* test was used for comparisons. ECs: endothelial cells; PDH-E1α: pyruvate dehydrogenase E1 alpha; LDHA: lactate dehydrogenase A; NF-200: neurofilament-200; MBP: myelin basic protein; SFI: sciatic functional index; 3PO: 3-(3-Pyridinyl)-1-(4-pyridinyl)-2-propen-1-one.


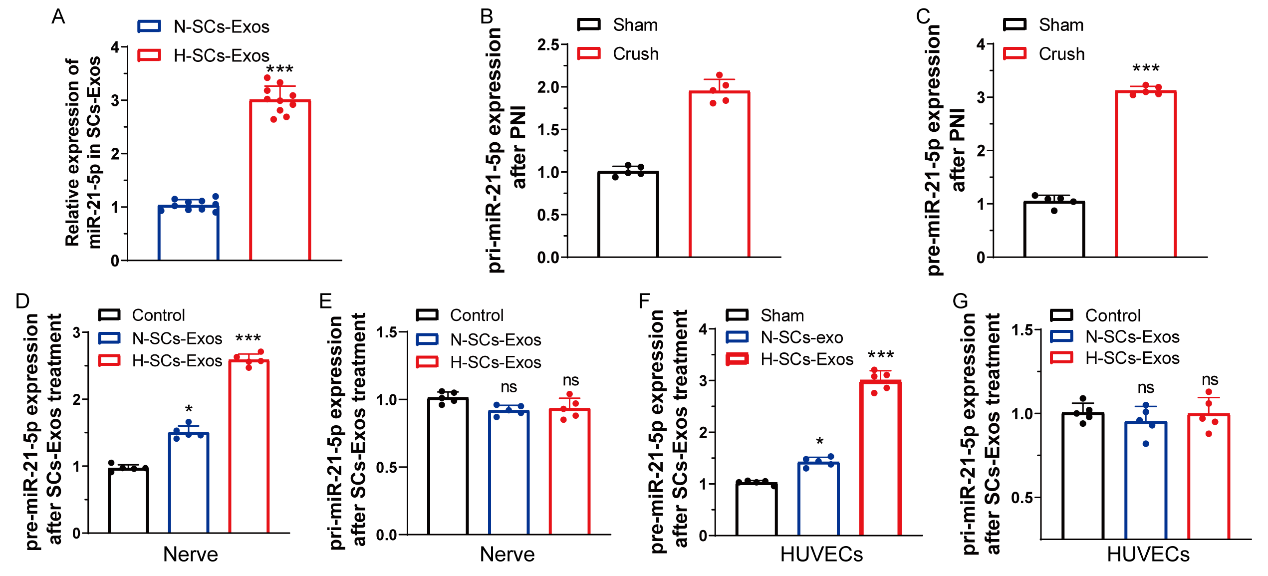


**Figure S5:** **H-SCs-Exos directly transferred miR-21-5p into ECs but didn’t stimulate its to produce new miR-21-5p.**

**A.** miR-21-5p content in H-SCs-Exos was significantly higher than that in N-SCs-Exos (*n* = 10 rats/group). **B.** pri-miR-21-5p expression and **C.** pre-miR-21-5p expression at injured segments of sciatic nerves (*n* = 5 rats/group). **D.** pri-miR-21-5p and **E.** pre-miR-21-5p expression at injured sciatic nerve segments after N-SCs-Exos or H-SCs-Exos administration (*n* = 5 rats/group). **F.** pri-miR-21-5p and **G.** pre-miR-21-5p expression in HUVECs after N-SCs-Exos or H-SCs-Exos administration (*n* = 5 rats/group).

^*^*P* < 0.05, ^**^*P* < 0.01, ^***^*P* < 0.001; *n* = 5 rats/group, Bars represent group means ± SD. A Student’s *t* test was used for comparisons. SCs: Schwann cells; HUVECs: human umbilical vein endothelial cells.


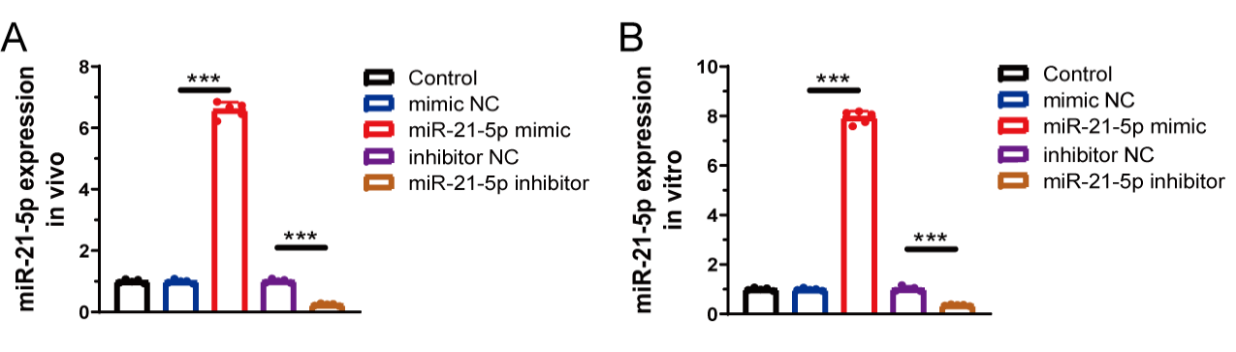


**Figure S6: Overexpression or knockdown of miR-21-5p (A) in vivo and (B) in vitro.**

^*^*P* < 0.05, ^**^*P* < 0.01, ^***^*P* < 0.001; *n* = 5 rats/group, Bars represent group means ± SD. A Student’s *t* test was used for comparisons. SCs: Schwann cells; HUVECs: human umbilical vein endothelial cells.


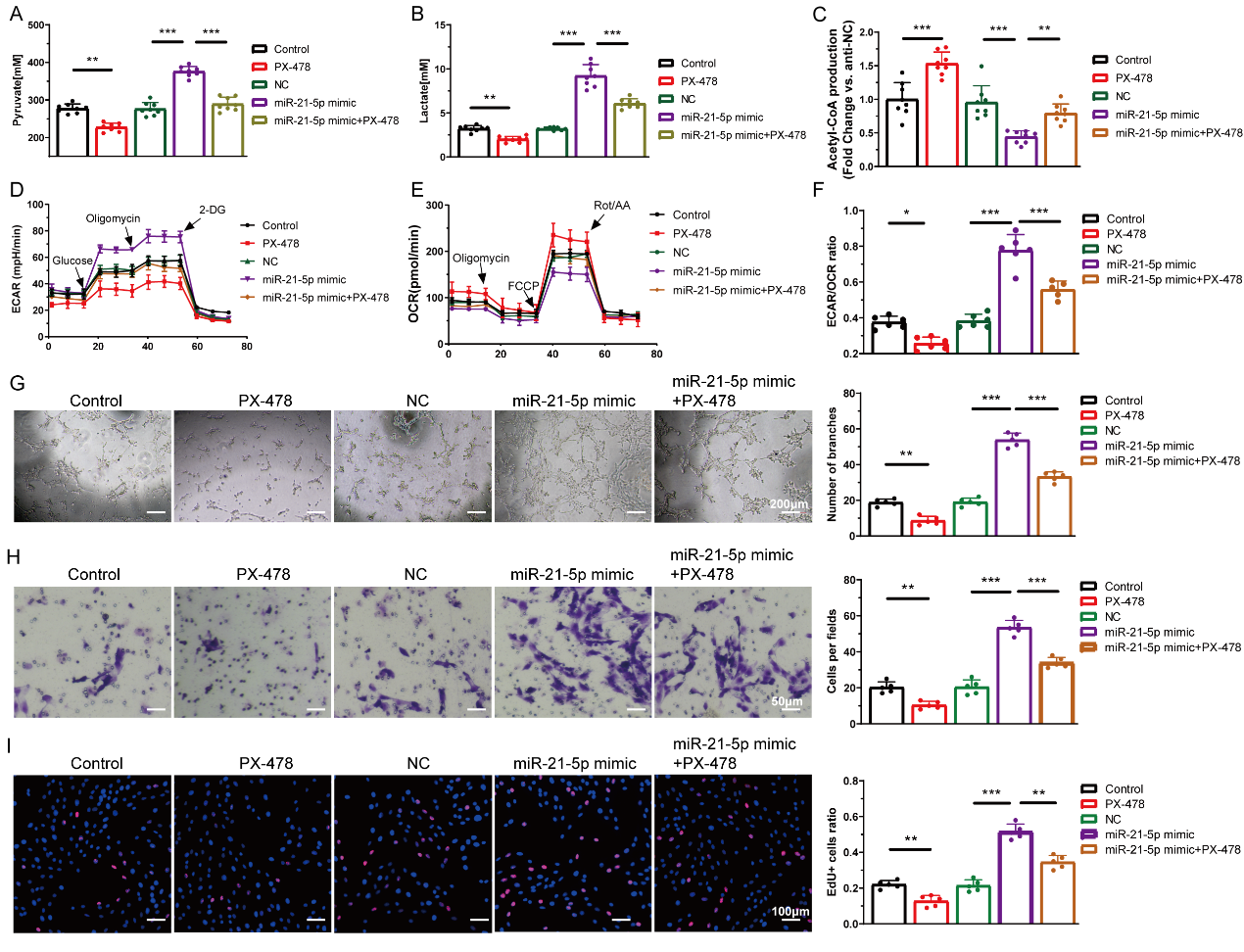


**Figure S7: miR-21-5p enhances glycolysis and inhibits mitochondrial respiration-related OXPHOS of ECs in vitro.**

**A.** Pyruvate production, **B.** lactate production, and **C.** acetyl-CoA production in HUVECs after PX-478 treatment or overexpression of miR-21-5p (*n* = 8 rats/group). **D.** ECAR assay and analysis including **E.** glycolysis, **F.** glycolysis capacity analysis, and **G**. glycolysis reserve analysis for evaluating glycolysis level of HUVECs after PX-478 treatment or overexpression of miR-21-5p (*n* = 6 rats/group). **H.** OCR assay and analysis including measurement of **I.** basal respiration, **J.** maximal respiration, **K.** spare respiration, and **L.** non-mitochondrial respiration for evaluating mitochondrial respiration of HUVECs after PX-478 treatment or overexpression of miR-21-5p (*n* = 6 rats/group).

^*^*P* < 0.05, ^**^*P* < 0.01, ^***^*P* < 0.001; *n* = 5 rats/group. Bars represent group means ± SD. A Student’s *t* test was used for comparisons. HUVECs: human umbilical vein endothelial cells; ECAR: extracellular acidification rate; OCR: oxygen consumption rate.


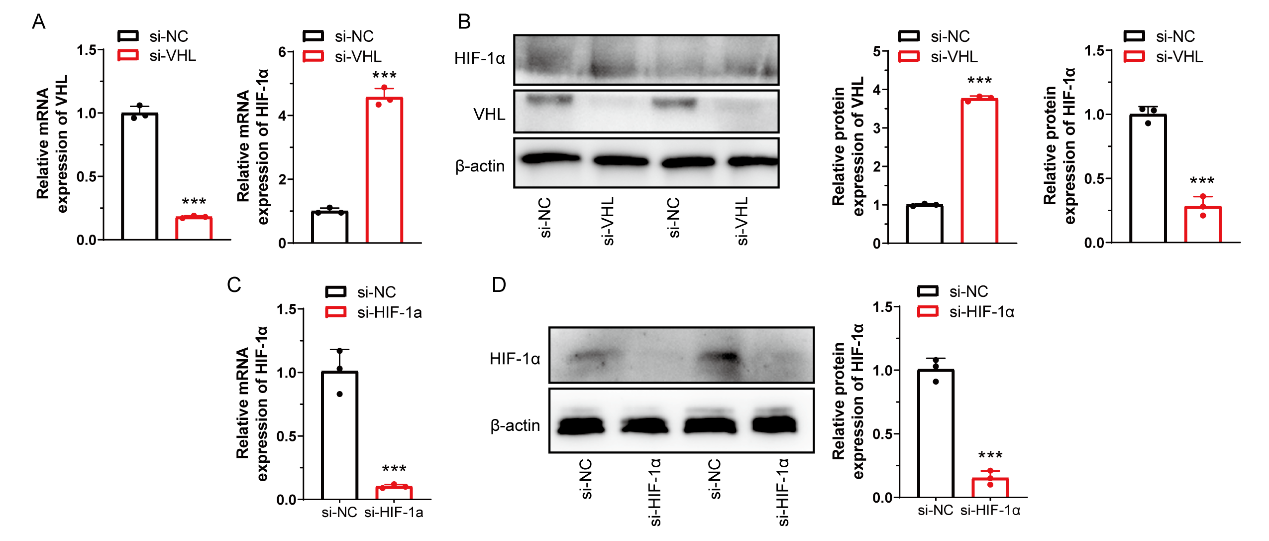


**Figure S8: Successful overexpression and knockdown of VHL and HIF-1α content in HUVECs.**

**A.** qRT-PCR assay for detecting mRNA expression of VHL and HIF-1α in HUVECs after knockdown of VHL with si-VHL (*n* = 3 rats/group). **B.** Western blot assay for detection of protein expression of VHL and HIF-1α in HUVECs after knockdown of VHL with si-VHL (*n* = 3/group). **C**. qRT-PCR assay for detecting mRNA expression of HIF-1α in HUVECs after knockdown of HIF-1α with si-HIF-1α (*n* = 3 rats/group). **D.** Western blot assay for detection of protein expression of HIF-1α in HUVECs after knockdown of HIF-1α with si-HIF-1α (*n* = 3 rats/group).

^*^*P* < 0.05, ^**^*P* < 0.01, ^***^*P* <0.001; *n* = 5 rats/group. Bars represent group means ± SD. A Student’s *t* test was used for comparisons. HUVECs: human umbilical vein endothelial cells; VHL: von Hippel-Lindau; HIF-1α: hypoxia-inducible factors 1 alpha.

**Table S1. Sequences of siRNAs and miR-21-5p mimics and inhibitors**

| Gene | Sequence |
| --- | --- |
| miR-21-5p mimic | 5′- CGCGGTAGCTTATCAGACTGATGTTGA -3' |
| miR-21-5p inhibitor | 5′-UCAACAUCAGUCUGAUAAGCUA-3' |
| miR-21-5p scramble | 5’-UUGUACUACACAAAAGUACUG-3’ |
| si-HIF-1α  si-VHL | 5'- GCATTGTATGTGTGAATTACG -3' |
|  | 5’- GGAATTGCAGCATATCGTT-3’ |
| si-PDK1 | 5’-CAAGCAACUGUCAUAUAUA-3’ |

**Table S2. Primer sequences**

| Gene | Direction | Sequence |
| --- | --- | --- |
| miR-21-5p | Forward | 5′- CGCGGTAGCTTATCAGACTGATGTTGA -3' |
|  | Reverse | 5′- GTGCAGGGTCCGAGGT -3' |
| VHL | Forward | 5′-AGGTCACCTTTGGCTCTTCA-3' |
|  | Reverse | 5′-ACATTTGGGTGGTCTTCCAG-3' |
| HIF-1α  PDH-E1α  PDK1 | Forward | 5'-AGGTGGATATGTCTGGGTTG-3' |
|  | Reverse  Forward  Reverse  Forward  Reverse | 5'-AAGGACACATTCTCTGTTT-3'  5′-CTTACCGCTACCATGGACACAGCATG-3'  5′-CTCCTTTAATTCTTCAACACTTGCAAGA-3'  5′-AAGAGCCCATCCTCTGTG-3'  5′-TGTTCATCTCGGAGCCTGTAG-3' |
| U6 | Forward  Reverse | 5′- CTCGCTTCGGCAGCACA -3'  5’-AACGCTTCACGAATTTGCGT-3’ |

**Table S3. Anti-bodies**

| Anti-bodies | | Manufacturers | Cat | Dilutions | Applications | |  |  |
| --- | --- | --- | --- | --- | --- | --- | --- | --- |
| Anti-CD63 | | Abcam | #ab252919 | 1:1000 | WB | |  |  |
| Anti-CD9 | | Abcam | #ab236630 | 1:1000 | WB | |  |  |
| Anti-TSG101 | | Affinity | #DF8427-100 | 1:1000 | WB | | |  |
| Anti-calnexin | | Abcam | #ab225542 | 1:1000 | WB | | |  |
| Anti-Glut1 | | Proteintech | #21829-1-AP | 1:2000 | WB | | |  |
| Anti-LDHA | | Cell Signaling Technology | #3582S | 1:1000 | WB | | |  |
|  | | | | 1:200 | IF | | |  |
|  | | | | 1:500 | IHC | | |  |
| Anti-PFKFB3 | | Proteintech | #13763-1-AP | 1:2000 | WB | | |  |
| Anti-HK2 | | Affinity | #BF0283-50 | 1:1000 | WB | | |  |
| Anti-HIF-1α | | Cell Signaling Technology | #14179s | 1:1000 | WB | | |  |
| Anti-VHL | Cell Signaling Technology | #68547S | 1:1000 | | WB | | |  |
| Anti-PDH-E1α | Proteintech | #18068-1-AP | 1:5000 | | WB | | | |
|  | | | 1:300 | | IF | | | |
|  | | | 1:500 | | IHC | | | |
| Anti-α-tubulin | Affinity | #T0033-50 | 1:5000 | | WB | | | |
| Anti-β-actin | Affinity | #T0022-100 | 1:5000 | | WB | | | |
| Anti-MBP | Cell Signaling Technology | #78896 | 1:50 | | IF | | | |
| Anti-NF-200 | Cell Signaling Technology | #30564 | 1:50 | | IF | | | |
| Anti-CD31 | Abcam | #ab182981 | 1:3000 | | IHC/IF | | | |
